# Southern Illinois Soybean Varieties: Implications for Relative Livestock Feed Value Scores, Modeled Soybean Meal Values, and Livestock Diet Formulation

**DOI:** 10.3390/ani16193105

**Published:** 2026-10-02

**Authors:** Jayakrishnan Nair, Richard Bien, Mrudhula Sajeevadathan, Talon Becker, Jebaraj Asirvatham, Kishore Joseph, John Osthus, Barton S. Borg, David Bishop, Mike Opperman, Amer AbuGhazaleh

**Affiliations:** 1 School of Agricultural Sciences, Southern Illinois University, Carbondale, IL 62901, USA; richard.bien@siu.edu (R.B.); jebaraj@siu.edu (J.A.); kishore.joseph@siu.edu (K.J.); aabugha@siu.edu (A.A.); 2 School of Medicine, Southern Illinois University, Carbondale, IL 62901, USA; mrudulajk@gmail.com; 3 Department of Crop Sciences, Illinois Extension, University of Illinois Urbana-Champaign, Champaign, IL 61822, USA; tbecker2@illinois.edu; 4 Blue Spring Ecosystem Services, 948 Imperial Pt., Manchester, MO 63021, USA; john@blue-spring.com; 5 Passel Farms, Main St. Emmetsburg, IA 50536, USA; bart.borg@passelfarms.com; 6 Reproductive Design Services, Pittsfield, IL 62363, USA; david@reprodesigns.com; 7 Black Dirt Communications, Fort Atkinson, WI 53538, USA; mike@blackdirtcommunications.com

**Keywords:** nutrient composition, backgrounding beef cattle, modeled SBM, livestock emissions

## Abstract

The research evaluated how location, crop year, relative maturity, and nutrient composition affect modeled soybean meal (SBM) values and the relative livestock feed value (LFV) scores of soybean varieties grown in southern Illinois. The study also assessed the impact of non-ruminant-specific LFV scores and modeled SBM composition in ruminant diets on requirements for other feed ingredients, such as feed corn. Among 47 soybean varieties collected from two southern Illinois locations over two growing seasons, modeled SBM values ranged from US$340.0 to US$370.0 per ton, while relative LFV scores varied from 1.3 to 9.9. SBM inclusion in the modeled backgrounding beef cattle diets ranged from 331.8 kg to 367.1 kg/ton, and feed corn inclusion ranged from 82.4 kg to 117.6 kg/ton. The feed formulation model showed a positive association for soybean seed protein with methane (CH_4_) emissions (r = 0.94 for CH_4_ as % GE, r = 0.98 for CH_4_ in mol/d, and r = 0.89 for CH_4_ in Mcal/d), though the relative range in predicted CH_4_ emissions from the feed formulations was only 1.8%, suggesting minimal impact of soybean varieties and limited practical significance. The feed formulation model showed a positive association between corn demand and dietary CP concentrations (*R*^2^ = 0.85). Meal from superior soybean seeds is predicted to be required at lower inclusions in model ruminant diets to achieve a net positive metabolizable protein balance.

## Simple Summary

The study aimed to evaluate the effects of soybean varieties, relative maturity groups, and crop years for Southern Illinois on nutrient composition and amino acid concentrations of seeds and modeled meals. Samples of 47 soybean varieties collected from two locations over two years were analyzed for CP and amino acid concentrations and ranked following the Illinois Soybean Association’s check-off-funded High Yield + Quality (HY + Q) program for non-ruminant-specific relative livestock feed value (LFV) scores, with 1 the lowest and 10 the highest. Higher crude protein and amino acid concentrations of modeled soybean meal (SBM) in general resulted in a better LFV score for the seeds. The CP and amino acid concentrations of the modeled SBM were incorporated into backgrounding beef cattle diets using the Beef Cattle Nutrient Requirement Model of the National Academies of Sciences, Engineering and Medicine. In the model backgrounding beef cattle diets, estimated SBM inclusion ranged from 331.8 kg to 367.1 kg (33.2 to 36.7%), and estimated feed corn inclusion ranged from 82.4 kg to 117.6 kg (8.2 to 11.8%) per ton of total feed when the diets were balanced for metabolizable protein concentrations. Higher CP and amino acid concentrations increased the LFV score of soybeans, resulting in an estimated decrease in inclusion of SBM and an increase in corn inclusion when backgrounding beef cattle diets were balanced for metabolizable protein concentrations. Moreover, estimated methane production metrics increased with increasing dietary CP concentrations.

## 1. Introduction

Soybeans are one of the major cash crops in the United States, with 80.4 million acres of harvested area yielding 4.26 billion bushels (127.8 million tons) of soybeans in 2025 [1]. Soybean cash receipts are forecasted at 44.2 billion dollars in 2025 [2]. The price paid to soybean farmers closely reflects the quality of the processed soybeans, including the value of soybean meals (SBM), oil, and hulls realized by processors. The crude protein (CP) concentrations, particularly the amino acid profile, significantly impact the SBM value when used in animal feeds. The checkoff-funded High Yield plus Quality (HY + Q) program [3] of the Illinois Soybean Association (ISA) targeted methods to identify soybean value at a variety level, facilitating opportunities for value improvement. The HY + Q program assigned soybean varieties a non-ruminant-specific relative livestock feed value (LFV) score ranging from 1 to 10, with 1 being the lowest and 10 the highest [4]. Soybean varieties with greater yield and superior CP and amino acid concentrations received higher LFV scores. The 2021 Illinois Soybean Variety Rankings, after evaluating more than 54,000 soybean samples, assigned LFV scores ranging from 2.0 to 9.3 for the 170 soybean varieties evaluated [4].

SBM is a major protein source for livestock, and its protein quality profoundly affects their growth performance. Poultry (60.7%), dairy (16.8%), and swine (16.3%) constitute the primary livestock sectors utilizing SBM, followed by beef cattle (3.6%) [5]. Soybean meal is the preferred protein source in poultry diets because of its rich nutrient composition and amino acid profile [6] while ruminants can effectively utilize alternative protein sources and non-protein nitrogen. As LFV scores were developed exclusively from non-ruminant nutrient parameters, their relevance to ruminant nutrition remains unclear. This knowledge gap is particularly important given the fundamental differences in protein and amino acid utilization between non-ruminants and ruminants, where ruminal fermentation and microbial protein synthesis substantially alter nutrient availability. Evaluating these non-ruminant-derived LFV scores in modeled beef cattle diets therefore provides a novel assessment of whether soybean quality rankings developed for swine and poultry translate into meaningful differences in SBM and feed corn inclusion and overall nutrient composition of ruminant diets. Moreover, the final report of the HY + Q program [7] indicated that adopting soybean varieties having a greater LFV score may likely inspire the processors to develop a new pricing structure based on the amino acid profile of the meal, which can, in turn, benefit the soybean farmers with greater opportunities for receiving higher prices for their seeds. We hypothesized that soybean varieties grown in southern Illinois vary in their LFV score, nutrient composition, and subsequent relative soybean meal values. We also hypothesized that selecting soybean varieties with higher LFV scores and relative soybean meal values will impact the inclusion of SBM and feed corn in ruminant diets. The objectives of the study included evaluating the LFV scores of soybean varieties grown in Southern Illinois and assessing the effects of incorporating SBM with varying feed value into ruminant diets on the requirements for other feed ingredients, such as feed corn.

## 2. Materials and Methods

### 2.1. Soybean Sampling

Soybean samples (~1.0 kg each) were collected from the University Farms (37° 42′05.2″ N, 89° 14′40.9″ W) of Southern Illinois University (SIU; Carbondale, IL, USA) under corn–soybean rotation, and from the variety trial experimental plots (38° 05′47.9″ N, 88° 50′58.4″ W) of Ewing Demonstration Center (Ewing, IL, USA) during the crop years 2022 and 2023. A total of seven seed varieties marketed by seed supplier 1 in 2022 and two varieties marketed by seed supplier 2 in 2023 were collected from the University farms of SIU. Similarly, from the Ewing Demonstration Center, a total of ten varieties marketed by seed supplier 3, eight varieties marketed by seed supplier 4, and six varieties marketed by seed supplier 5 were collected in 2022, while four varieties marketed by seed supplier 2 and ten varieties marketed by seed supplier 3 were collected in 2023. There were a total of 5 seed suppliers and 47 varieties across two crop years. The relative maturity of seeds ranged from 3.0 to 4.9 across locations and years. Seeds were harvested using a plot combine. Seeding, management, and harvest practices were similar across varieties for each location and crop year.

### 2.2. Climatic Conditions

Environmental conditions, including maximum, minimum, and mean temperature and cumulative precipitation data, were collected from the weather stations at Southern Illinois University Farm and Ewing Demonstration Center during both crop years.

### 2.3. Chemical Analysis

Individual soybean samples (~1.0 kg) collected were mixed well, and subsamples (~50 g) were sent to the Agricultural Experiment Station Chemical Laboratories (ESCL) of the University of Missouri (Columbia, MO, USA) for analysis according to the Official Methods of Analysis of the Association of Official Analytical Chemists (AOAC) [8]. Samples were ground at ESCL and were analyzed for dry matter (DM; AOAC 934.01), CP (AOAC 990.03), and complete amino acid profile (AOAC 982.30). Individual samples were subjected to single-run analysis for DM, CP, and amino acid concentrations using cation-exchange chromatography coupled with post-column ninhydrin derivatization and quantitation.

### 2.4. Estimation of Relative Soybean Meal Values

Whole soybean CP and amino acid concentrations were converted into a “virtual meal” or “modeled SBM” nutrient composition following the modeling assumptions of 12% seed moisture content and 74% meal yield [9,10]. The composition of each modeled meal was incorporated into least-cost ration formulations for swine (nursery, finishing, and lactation) and poultry (broiler and layer), accounting for SBM use, total diet costs (US$/ton), and current market ingredient prices (US$/ton) based on the USDA historical WASDE report data [11,12]. The soybean variety with the highest cost for SBM ($/ton of diet) in the respective diets was assigned a base value of US$340/ton for SBM as a modeling assumption based on historic HY + Q soybean meal valuation [13] to compare soybean meal values across the individual soybean samples. The soybean meal value for each variety was calculated as the SBM requirement for that variety in the diets, relative to the base sample composition, and accounted for essential amino acid concentrations (lysine, methionine, threonine, and tryptophan), dietary energy, and standardized ileal digestibility coefficients. High concentrations of these amino acids reduced the need for producers to purchase expensive synthetic amino acids, thereby lowering feed costs and increasing the value of soybean meals. To estimate the relative soybean meal values, the difference in formula cost was applied to the base price based on the amount of SBM used per ton. The price difference may reflect the quantity of SBM, or other feed ingredients used in the feed formulation while maintaining the required levels of nutrients and energy in the diets of livestock species and production stages [11].

### 2.5. Estimation of Relative Livestock Feed Value Scores of Soybean Varieties

Relative livestock feed value scores were assigned to soybean varieties following the procedure employed by the HY + Q program before the Dalex software (CN. Dalex, The Consulting Nutritionist, Version 3, Dalex Livestock Solutions LLC, Bloomington, MN, USA) integration [14]. Briefly, the relative soybean meal values were evaluated as described previously. Soybean varieties were ranked based on their ability to provide the most economical, high-quality amino acid composition for the classes of livestock and the physiological stage of growth (swine—nursery, finishing, and lactation, and poultry—broiler and layer). The variety requiring the smallest quantity in the diet formulation to meet the amino acids, nutrients, and energy requirements of the livestock species received the highest relative livestock feed value score, while the variety requiring the highest inclusion in livestock diets received the lowest LFV score among the 47 samples evaluated.

### 2.6. Soybean Meal Value in Ruminant Diets

The composition of the modeled SBM, as described above, was used to evaluate the effects of soybean variety nutrient composition on inclusion levels in ruminant diets. The concentrations of protein and amino acids of individual SBM were included in the diet formulation using the 2016 Beef Cattle Nutrient Requirements Model (BCNRM, 2016) [15] of the National Academies of Sciences, Engineering, and Medicine (NASEM, 2016) [16] for beef cattle diets. Diets were formulated for implanted backgrounding beef cattle (Angus × Simmental) weighing 300 to 400 kg BW and gaining at 1.2 kg/d. The model diets (50:50 forage:concentrate) consisted of 40% corn silage, 10% bromegrass hay, 45% feed corn + SBM, and 5% vitamin–mineral supplement (% DM basis). The model diets were formulated to meet or exceed the NASEM (2016) nutrient requirements for the targeted growth level. Calcium-to-phosphorus ratios were formulated to range from 1.5:1 to 2:1. The level of inclusion of corn silage, bromegrass hay, and vitamin–mineral supplement was fixed, while the level of inclusion of feed corn changed with the inclusion of each SBM from individual varieties varying in protein concentrations and amino acid profiles. The amino acid composition of all 47 modeled SBMs was entered into the feed formulation, as SBM was the primary protein source in the model diets, and the amino acids from the other feed ingredients were similar due to comparable inclusion levels across diets. The diets were formulated to have a net positive RUP supply and metabolizable protein balance.

### 2.7. Calculations and Statistical Analysis

Soybean yields in bu/ac are converted to kg/ha by multiplying the bushel weight by 67.25 [17], as one bushel (60 lb. bushel) of soybean is 27.22 kg and one hectare is 2.47 acres. Distinct statistical models were employed for each primary factor of interest. In the relative maturity (RM) analysis, RM category, crop year, and location were treated as categorical fixed effects to estimate and compare least squares means (LS-means) among RM categories. Crop year (2022 and 2023) and location (Carbondale and Ewing) were included to address temporal and site-specific variation, since observations within each RM category spanned multiple crop years and locations. Consequently, the LS-means for RM categories represent values adjusted for differences in crop year and location.

In analyses of crop year and location, we used the observed RM value for each observation as a continuous covariate rather than RM category. This adjustment accounted for variation in actual RM among observations when estimating LS-means for crop year and location. Here also, we interpret differences among crop years, and locations as adjusted associations rather than evidence of causal effects.

For the relative SBM and LFV analyses, we used separate one-way categorical models to estimate means for low, medium, and high categories. Because SBM and LFV categories were derived from soybean composition, the resulting *p*-values indicate statistical differences in the response variable among the derived categories, and results should not be interpreted as evidence of independent causal effects. LS-means, standard error of the mean (SEM), and *p*-values are reported for all above models.

Supplier effects are presented descriptively, with potential confounding by year and location considered when interpreting the results.

The data were analyzed using SAS (version 9.4; SAS Institute Inc., Cary, NC, USA) [18] PROC MIXED with the following models:

RM Analysis:

*Y_iljk_* = *μ* + *RM_l_* + *Year_j_* + *Location_k_* + *ε_iljk_*,

where *Y_iljk_* is the measured trait for observation *i* belonging to RM category *l*, crop year *j*, and location *k*, *μ* is the overall mean, *RM_l_*, *Year_j_* and *Location_k_* are the fixed effects being estimated, and *ε_iljk_* is the residual error.

Crop Year and Location Analyses:

*Y_ijk_* = *μ* + *RM_ijk_* + *Year_j_* + *Location_k_* + *ε_ijk_*,

where *Y_ijk_* is the measured trait for observation *i* belonging to crop year *j*, and location *k*, *μ* is the overall mean, *RM_ijk_* is the continuous covariate, *Year_j_*, and *Location_k_* are the fixed effects being estimated, and *ε_ijk_* is the residual error.

Relative SBM and LFV Analyses:

*Y_ij_* = *μ* + *C_j_* + *ε_ij_*,

where *Y_ij_* measured trait for observation *i*, *μ* is the overall mean, *C_j_* is the fixed effect of the derived relative SBM or LFV category (low, medium, high), and *ε_ij_* is the residual error.

The denominator degrees of freedom were determined using the Kenward–Roger option. Mean separation was carried out by Tukey’s test. The PROC CORR procedure in SAS was used to evaluate the Pearson correlation between the variables. Significant differences and trends were declared at *p* < 0.05 and *p* < 0.10, respectively.

## 3. Results

### 3.1. Soybean Samples

The soybean varieties evaluated in the study varied across locations and crop years (Table 1). A total of 9 samples were collected from Carbondale, and 38 from the Ewing location during 2022–2023. Of the 10 seed varieties marketed by supplier 3 and collected from the Ewing location in 2023, 6 were the same as in 2022 from the same location.

### 3.2. Soybean Relative Maturity Groups and Nutrient Composition

Relative maturity of soybean varieties ranged from 3.3 to 4.9 in year 1 and from 3.0 to 4.8 in year 2 across locations (Table 2). Soybean yield averaged 48.3 ± 9.78 bu/ac while the seed DM averaged 91.7% ± 1.39% and seed protein concentration averaged 40.1% ± 1.31% across varieties, locations, and crop years (Table 2). Of the 47 soybean varieties evaluated, 10 varieties (2 from Carbondale and 8 from Ewing) had RXF technology (i.e., Roundup Ready 2 XtendFlex), which provides resistance to dicamba, glyphosate, and glufosinate, while the remaining 37 varieties had E3 technology, indicating herbicide tolerance to 2,4-D choline, glyphosate, and glufosinate. Further, 19 varieties were sulfonylurea-tolerant while 28 were not, and 39 had good soybean cyst nematode resistance across varieties, locations, and crop years. As the soybean varieties (*n* = 47) evaluated in the present study belonged to relative maturity groups 3.0 to 4.9, the varieties were grouped into four relative maturity groups (relative maturity ranging from 3.0 to 3.5, 3.6 to 4.0, 4.1 to 4.5, and 4.6 to 5.0) for further comparative evaluation (Table 3). There were 10 varieties in maturity group 3.0–3.5, 14 in group 3.6–4.0, 13 in group 4.1–4.5, and 10 in group 4.6–5.0. Soybean CP, amino acid concentrations, or relative soybean meal values did not vary between relative maturity groups. Soybean yield (bu/ac and kg/ha) was numerically greater for the relative maturity group 3.6–4.0, while the yield remained similar for the other relative maturity groups.

### 3.3. Relative Soybean Meal Values

Across soybean varieties, locations, and years, the relative soybean meal values ranged from US$340.0 to US$365.5 in swine nursery diets, US$340.1 to US$364.6 in swine finishing diets, US$343.3 to US$368.9 in swine lactation diets, US$340.1 to US$370.0 in broiler diets, and US$340.2 to US$368.3 in layer diets (Table 4). The relative soybean meal values ranged from US$349.7/ton to US$367.3/ton in year 1 and from US$341.0 to US$361.8/ton in year 2 across varieties and locations. Soybean varieties with varying relative soybean meal values were categorized into High-value (≥US$360.1/ton), Medium-value (US$350.1–US$360.0/ton), and Low-value (≤US$350.00/ton) categories across varieties and locations for further comparative evaluation. There were a total of six soybean varieties in the Low-value category, 31 varieties in the Medium-value category, and 10 varieties in the High-value category (Table 5). All three categories of relative soybean meal values included soybean varieties from all four soybean relative maturity classes. The DM, CP, and amino acid concentrations, yield, and relative soybean meal values of High-value soybean varieties were greater (*p* ≤ 0.03) than those of Low-value varieties, with Medium-value varieties in between.

### 3.4. Relative Livestock Feed Value Scores of Soybean Varieties

**Figure 1:**
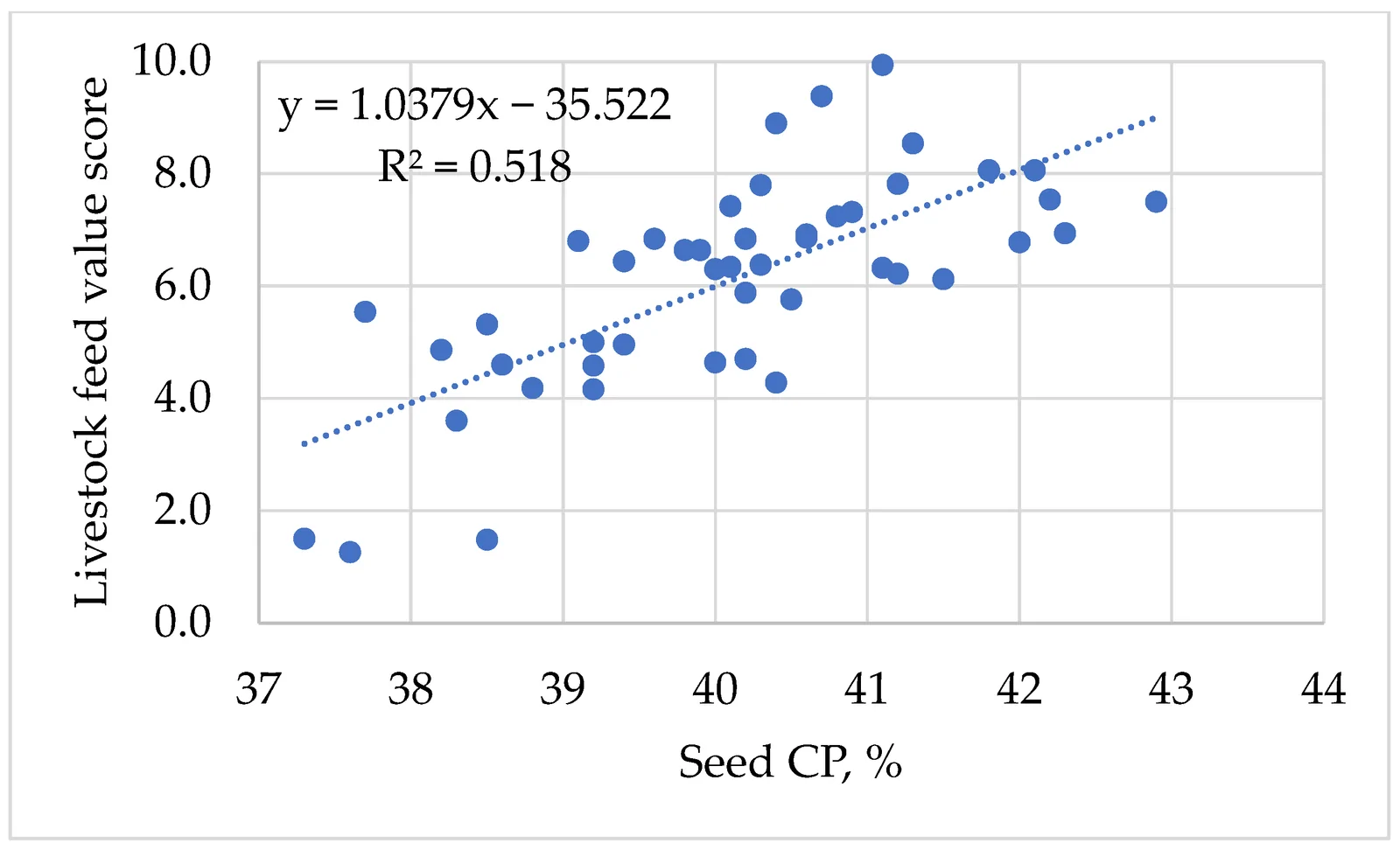
Association between soybean seed CP concentrations and livestock feed value scores.

The relative livestock feed value scores of soybean varieties ranged from 1.3 to 9.9 across locations and years, with year 1 samples from 4.2 to 9.9 and year 2 samples from 1.3 to 8.1 across locations (Table 6). The LFV scores reported above represent rankings among the 47 southern Illinois soybean samples collected from two locations during 2022–2023 for the present study. Caution should be exercised when comparing these scores with the HY + Q LFV scores reported [4]. In particular, the highest LFV score observed in the present study (9.9) represents a single sample among the 47 samples evaluated and should not be interpreted as representative of soybean samples across Illinois. The LFV scores of soybean varieties across the five rations (swine nursery, finishing, and lactation, and poultry broiler and layer) were averaged to obtain the average LFV score for all 47 soybean varieties evaluated. Overall, soybean varieties with greater CP concentration had higher (*R*^2^ = 0.52) LFV scores (Figure 1). The present study categorized the LFV scores of the soybean varieties into High-score (7.1–10), Medium-score (4.1–7.0), and Low-score (1.0–4.0) across varieties and locations for further comparative evaluation. There was a total of four soybean varieties under the Low-score category, 30 varieties under the Medium-score category, and 13 varieties under the High-score category (Table 7). The DM concentrations were numerically greater for High- than for Low-value soybeans. As expected, the CP and amino acid concentrations, yield, and relative soybean meal values were greater (*p* ≤ 0.01) for High- than for Low-value SBM with Medium-value varieties in between.

### 3.5. Environmental Conditions During Soybean Growth

The weather conditions during the soybean growth period (May–November in each year) in the 2022 and 2023 crop years are presented in Table 8. Maximum, minimum, and average temperatures during both crop years were similar across locations. Cumulative precipitation was relatively lower in 2022 vs. 2023 [370.6 mm (14.6 inches) vs. 540.3 mm (21.3 inches)] at Carbondale, while it was similar across the two crop years [431.3 mm (17.0 inches) in 2022 vs. 467.6 mm (18.4 inches) in 2023] and averaged 449.5 ± 25.7 mm (17.7 ± 0.99 inches) in Ewing. On average, the cumulative precipitation was lower in 2022 and greater in 2023 in Carbondale than in Ewing.

### 3.6. Crop Year and Nutrient Composition of Soybean

The relative maturity of soybean varieties ranged from 3.3 to 4.9 in 2022 and 3.0 to 4.8 in 2023 across locations and seed suppliers (Table 9). Soybean yield varied across crop years, with the 2022 yield greater than in 2023 (*p* < 0.0001). Soybeans sampled in the 2022 crop year had greater (*p* ≤ 0.01) DM and CP concentrations (Table 9) relative to 2023 samples. Relative soybean meal values (US$/ton) for soybean samples were greater (*p* < 0.0001) in 2022 than in 2023. Concentrations of most of the amino acids evaluated, including cysteine, isoleucine, lysine, and threonine, were also greater (*p* ≤ 0.02) for soybean samples in 2022 than in 2023.

### 3.7. Location and Nutrient Composition of Soybean

A total of 9 samples were collected from Carbondale and 38 from Ewing over two crop years (Table 10). The relative maturity of soybeans ranged from 3.3 to 4.9 in Carbondale and 3.0 to 4.8 in Ewing across years and seed suppliers. The DM concentration of soybeans was greater (*p* < 0.0001) for Ewing than for Carbondale across crop years. The location (Carbondale vs. Ewing) did not (*p* > 0.05) impact the yield, CP, amino acid concentrations except for lysine, or relative soybean meal values (Table 10).

### 3.8. Soybean Seed Suppliers and Nutrient Composition of Soybean

There were a total of 7 varieties from seed supplier 1, 6 from seed supplier 2, 20 from seed supplier 3, 8 from seed supplier 4, and 6 from seed supplier 5 across crop years and locations (Table 11). The relative maturity of varieties marketed by the seed suppliers spanned all four groups, ranging from 3.0 to 4.9. The DM content of soybean seeds from suppliers 4 and 5 (both in 2022 at the Ewing location) averaged 93.0%, while that of soybean varieties from supplier 1 (in the Carbondale location in 2022) averaged 90.1%, with suppliers 2 and 3 in between. Soybean yield (bu/ac) was relatively greater for varieties from seed suppliers 1 (49.7 ± 6.92), 4 (56.0 ± 2.78), and 5 (51.6 ± 3.26), relative to suppliers 2 and 3, averaging 38.6 ± 2.89 and 42.7 ± 6.46 bu/ac (2595.9 to 3768.0 kg/ha). The CP concentrations (% DM basis) of soybean varieties from suppliers 4 (41.8 ± 0.76) and 5 (40.8 ± 0.70) were numerically higher than those from suppliers 1 (39.6 ± 0.69), 2 (38.8 ± 1.25), and 3 (39.8 ± 1.10). Relative soybean meal values (US$/ton) were generally higher (US$358.8 ± 2.46/ton vs. US$350.4 ± 4.96/ton) for seeds from supplier 4 than for those from supplier 2 with others in between. Concentrations of amino acids, including isoleucine, lysine, threonine, and valine, were relatively greater for seeds from suppliers 4 and 5 than those from suppliers 1, 2, and 3.

### 3.9. Relative Soybean Meal Values for Ruminant Diets

**Figure 2:**
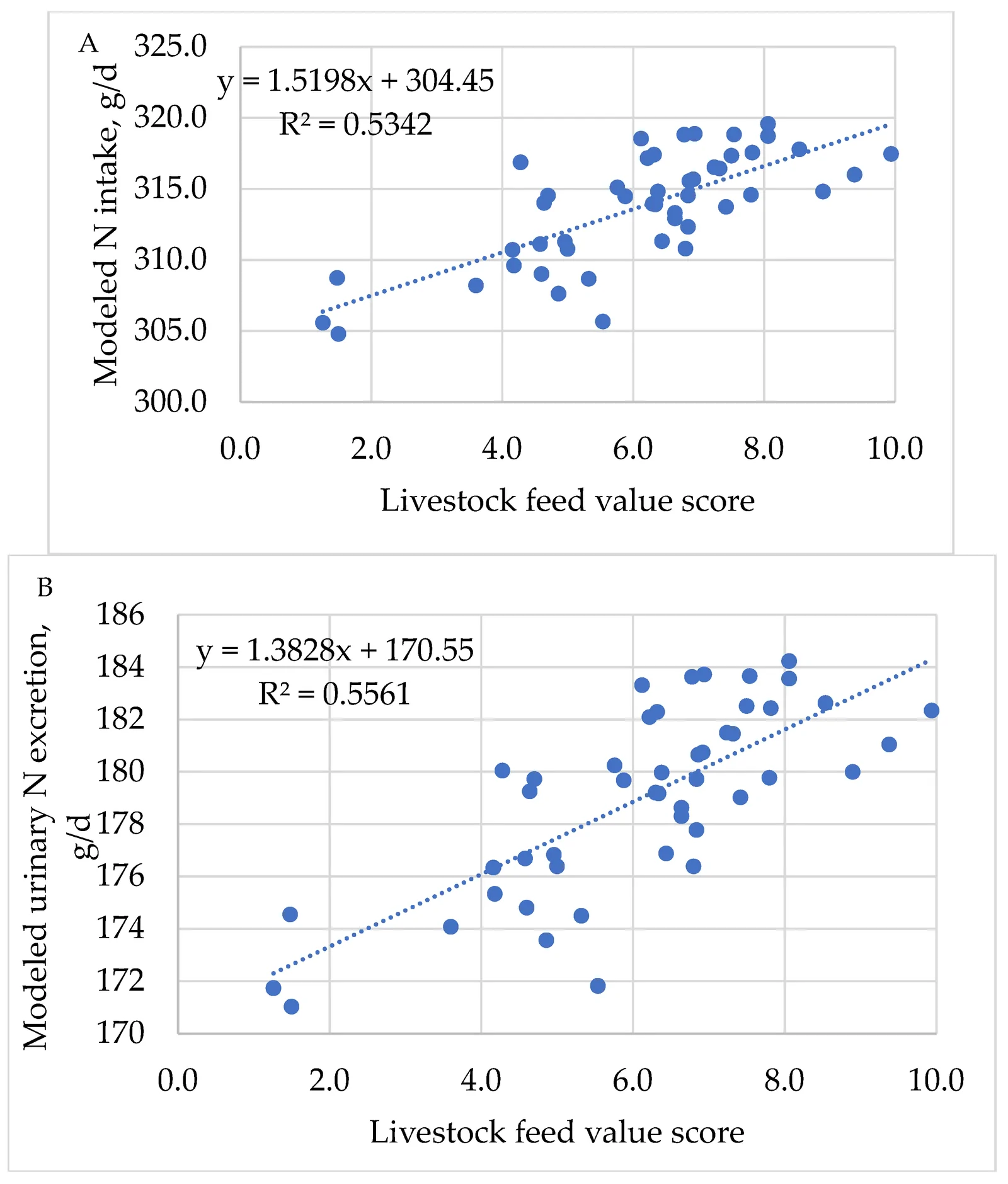
Association between (**A**) livestock feed value score of soybeans and estimated N intake (g/day) on feeding modeled diets to backgrounding steers and (**B**) livestock feed value score of soybeans and estimated urinary N excretion (g/day) on feeding modeled diets to backgrounding steers.

**Figure 3:**
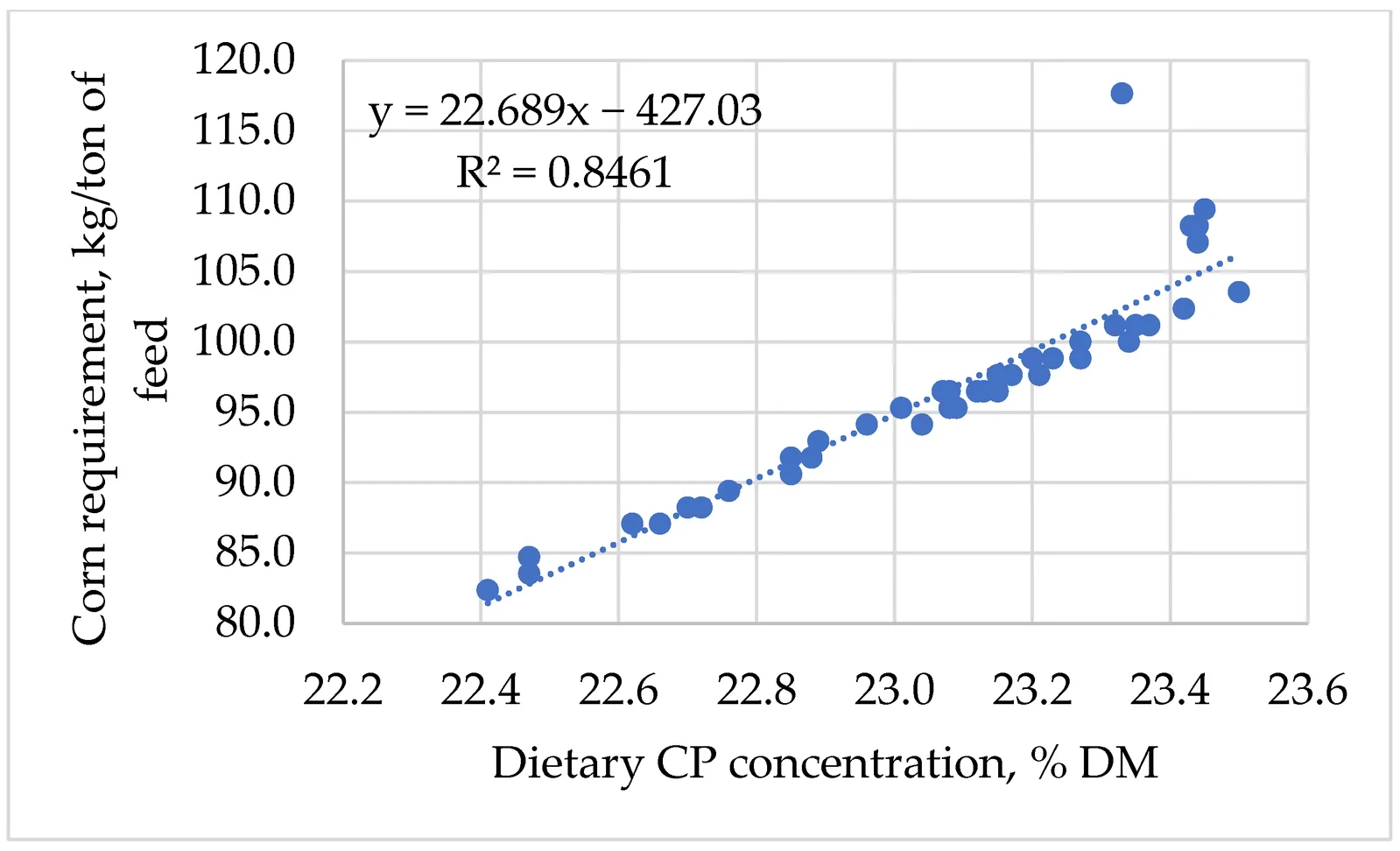
Association between dietary CP concentrations and corn requirement (kg corn/ton of feed).

**Figure 4:**
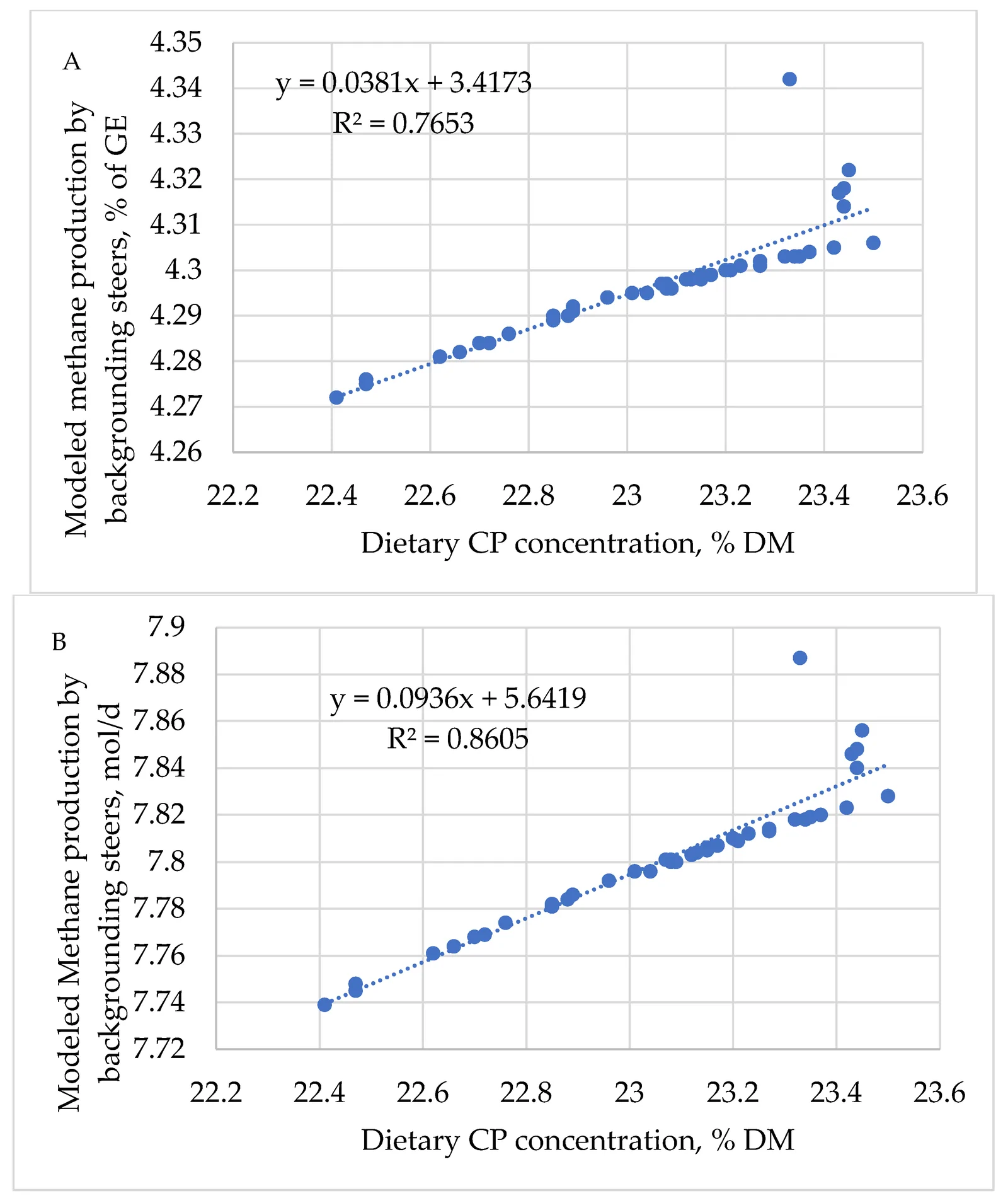

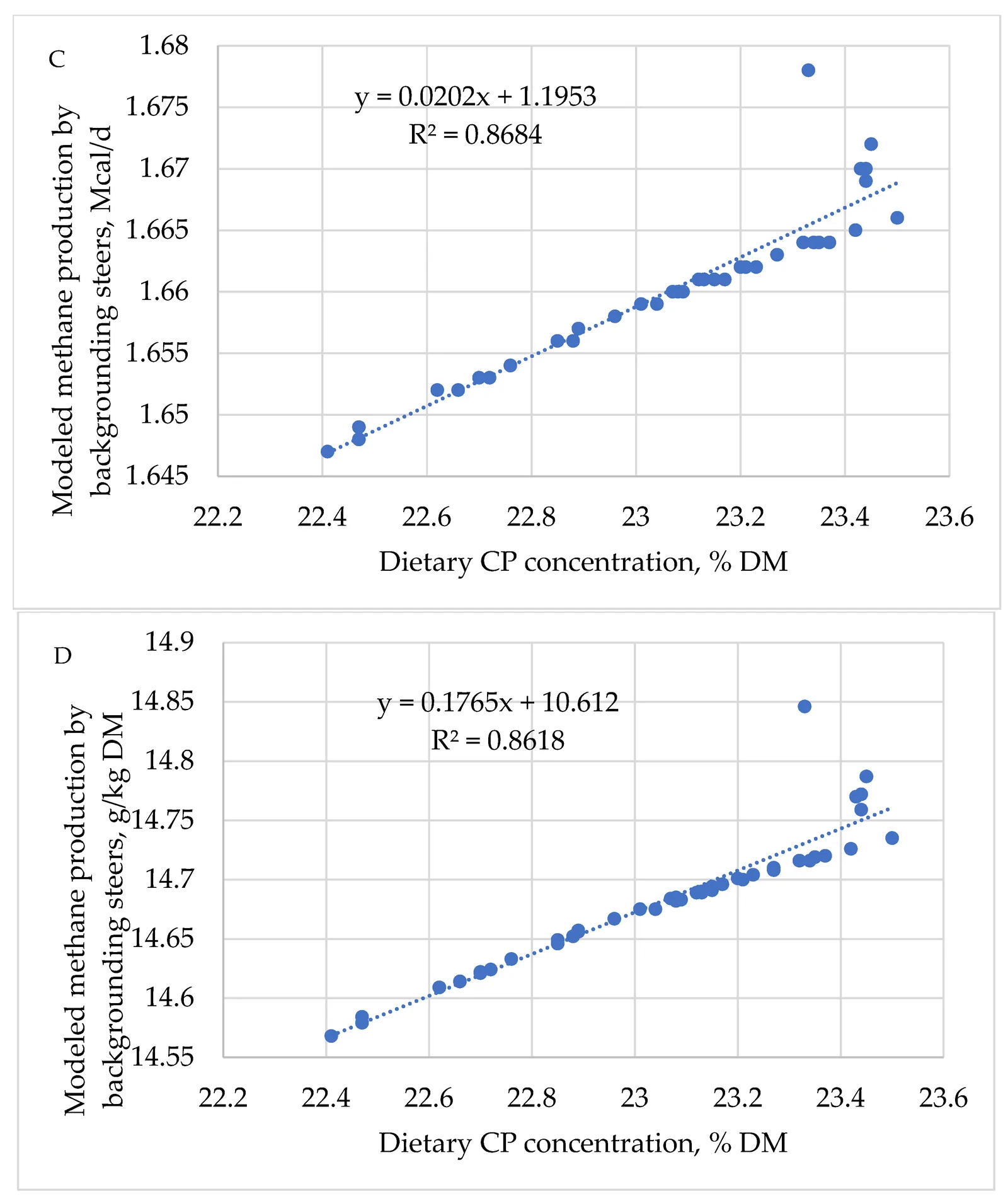
Association between dietary CP concentrations and (**A**) CH_4_ production as a % of gross energy (GE), (**B**) CH_4_ production in mol/d, (**C**) CH_4_ production in Mcal/d, and (**D**) CH_4_ production in g/kg DM.

The ingredient and nutrient composition of the model diet for backgrounding beef cattle are presented in Table 12. When the diets were formulated to have a net positive MP content (g/d) by incorporating the CP and amino acid concentrations of individual SBM into ration formulation, the inclusion of SBM varied from 331.8 kg (33.2% inclusion) to 367.1 kg (36.7% inclusion), while that of feed corn ranged from 82.4 kg (8.2% inclusion) to 117.6 kg (11.87% inclusion) per ton of the total mixed diet. A greater livestock feed value score for soybeans estimated an increase in N intake (g/day) on feeding modeled diets to backgrounding steers (*R*^2^ = 0.53). Similarly, greater livestock feed value score for soybeans estimated an increase in urinary N excretion (g/day) on feeding modeled diets to backgrounding steers (Figure 2A,B). Corn demand increased as SBM inclusion decreased in the modeled diets, resulting in lower dietary CP concentrations (Figure 3). The range in CH_4_ production metrics (4.27 to 4.34% of GE with 1.6% relative range, 7.74 to 7.89 mol/d with 1.9% relative range, 1.648 to 1.678 Mcal/d with 1.8% relative range, and 14.57 to 14.85 g/kg DM with a1.9% relative range) was minimal. The model predicted that greater CP concentrations in modeled diets were associated with increased predicted enteric CH_4_ emission across all evaluated metrics (CH_4_ production as a % of gross energy (GE) (Figure 4A), CH_4_ production in mol/d (Figure 4B), CH_4_ production in Mcal/d (Figure 4C) and CH_4_ production in g/kg DM (Figure 4D) (*R*^2^ = 0.77, 0.86, 0.87, and 0.86, respectively).

The descriptive Pearson correlation of soybean seed protein concentrations, SBM feed value, dietary composition, nitrogen balance, and enteric CH_4_ emissions from backgrounding beef cattle is presented in Table 13. Data from the current study samples indicated that TMR CP was positively associated with seed protein concentrations (*r* = 0.97), and CH_4_ emission metrics (*r* = 0.94 for CH_4_ as a % of GE, *r* = 0.98 for CH_4_ in mol/d, *r* = 0.89 for CH_4_ in Mcal/d, except for CH_4_ in g/kg DM, *r* = 0.08) while negatively associated (*r* = −0.98) with SBM inclusion in diets (kg/d). TMR CP was found to be positively associated with TMR starch (*r* = 0.91), CH_4_ emission metrics (*r* = 0.85 for CH_4_ as a % of GE, *r* = 0.91 for CH_4_ in mol/d, *r* = 0.81 for CH_4_ in Mcal/d, except for CH_4_ in g/kg DM, *r* = 0.08) and soybean meal value (*r* = 0.74) while negatively associated with SBM inclusion (*r* = −0.91). Similar to TMR CP, TMR starch (indicating feed corn inclusion) was found to be positively associated with CH_4_ emission metrics (*r* = 0.96 for CH_4_ as a % of GE, *r* = 0.99 for CH_4_ in mol/d, *r* = 0.91 for CH_4_ in Mcal/d, except for CH_4_ in g/kg DM, *r* = 0.08) and soybean meal value (*r* = 0.67). Retained N was not associated (−0.37 < r < 0.25) with any of the parameters evaluated. Methane emission metrics were negatively associated (*r* ranging from −0.92 to −0.99) with SBM inclusion in the diet. Soybean meal value was positively associated with Seed CP (r = 0.72), TMR CP (r = 0.74), and CH_4_ emission metrics (*r* = 0.63 for CH_4_ as a % of GE, *r* = 0.68 for CH_4_ in mol/d, *r* = 0.58 for CH_4_ in Mcal/d, except for CH_4_ in g/kg DM, *r* = 0.04).

## 4. Discussion

The major end product of soybean processing is the meal. The greater the value of SBM, the greater the harvest price the producers should receive. It is widely accepted that a higher CP concentration, specifically the essential amino acid profiles of soybeans, should result in higher prices for farmers, as high-quality SBM will increase demand for meals. This will result in processors receiving more value, allowing them to pay farmers more. To ensure the maximum cash price, the farmers must maximize the compositional quality of their soybeans. Given the interaction between soybean genetics and environmental effects (discussed below) on soybean varieties and their resultant meal composition, selecting soybean varieties that yield meal with desirable composition without any yield penalty is critical. Selecting soybean varieties that yield the highest-quality SBM will equip US soybean producers to compete for price premiums from soybean seed processors.

### 4.1. Environmental Conditions and Soybean Quality

Although environmental conditions, including maximum, minimum, and mean temperatures, as well as cumulative precipitation, were comparable, yield and nutrient composition, including concentrations of cysteine, isoleucine, lysine, and threonine, varied considerably between years. Significant spatial and interannual variability in soybean yield and composition has been reported across harvested areas in the United States [19]. A recent study conducted in Nebraska [20] quantified the CP and oil concentrations of soybean samples collected across multiple locations. Their results indicated that protein concentration averaged 34% and ranged from 31% to 36% across fields, with an in-field coefficient of variation (CV) of 3.3% (1.7–5.2%). Oil concentration averaged 20% across fields, ranging from 18% to 22%, with an in-field CV of 3.6% (2.1–6%) [20]. Soybean variety selection, environmental growing conditions such as temperature and precipitation, soil fertility, and mineral concentrations impact soybean seed yield and composition. Mourtzinis et al. [21] reported that greater temperatures during soybean seed filling reduced protein and increased oil concentrations. Further, irrigation was reported to alter soybean seed protein and oil content.

Guo et al. [22] reported significant variation in protein concentration in soybean germplasm. These authors also reported that soybean protein concentration varies significantly under different growing conditions. Soybean variety Cv. Williams has been reported to have protein concentrations between 32.8% and 48.7% when planted in 186 locations in 76 countries [23,24]. Similarly, in an evaluation of 13 soybean genotypes released between 1980 and 2014, Reis et al. [25] reported that seed yield increased by 50% and protein decreased by 1.2% when comparing the oldest and newest genotypes. These authors also reported that concentrations of amino acids such as leucine, proline, cysteine, and tryptophan were not influenced by genotype, while the concentrations of other amino acids showed a linear decrease over time. It should be noted that the soybean varieties were not the same between years across locations in the present study. The inherent variability in the interactions of selected soybean varieties with edaphic and environmental factors likely affected yield and nutrient composition each year. Variability in CP concentrations and corresponding variability in essential amino acid concentrations considerably affected the SBM meal value, which varied by about US$18.8/ton across livestock feed value score categories. The variability in soybean seed composition, in turn, can affect the SBM composition and the meal value. Variability in SBM values will affect the price per bushel offered locally, regionally, and internationally [26].

### 4.2. Soybean Seed Protein Concentrations and Relative Livestock Feed Value Scores of Soybean Varieties

The value of SBM varied by 5.5% (US$18.8), and the CP concentration varied by 8.7% (3.3-unit CP) between samples in low and high LFV score categories. Lower CP and proportionately lower amino acid concentrations decreased the LFV score of soybeans. The average soybean protein concentration decreased by 4.9% from 1986 to 2024, while the oil concentrations increased by 7.0% and yield increased by 59.6% during the same period [27]. This reduction in protein concentrations is mainly due to increased soybean production in northwestern regions with low protein concentrations and to newer varieties having intrinsically lower protein concentrations [28,29,30]. The major determinants of variability in regional-scale soybean seed composition are water availability, temperature, and soil fertility [31,32,33,34]. However, it should be noted that there is more substantial variation within than between soybean-producing regions. Brumm and Hurburgh [35] reported that the variability in CP concentration within a region (such as the western corn belt, eastern corn belt, mid-south, southeast, and east coast) is three times that observed between regions. Seed protein, specifically amino acid concentrations, impacts SBM quality and the subsequent growth and productivity of livestock species and the profitability of livestock producers.

The decline in soybean seed protein and, subsequently, SBM-amino acid concentrations has resulted in a significant reduction in the inclusion of SBM in livestock diets (swine and poultry), with the majority of SBM being replaced by synthetic amino acids and corn byproducts. This increased use of substitute feed ingredients at the expense of SBM translates to substantial losses in soybean sales and market share, in domestic and international livestock markets [36,37]. The HY + Q program facilitated the assignment of LFV scores for more than 170 soybean varieties marketed by 21 seed suppliers, based on more than 54,000 soybean samples evaluated [4]. It should be noted that the soybean variety rankings – Illinois [4] is the published HY + Q variety ranking brochure compiled from entries in the 2019 and 2020 F.I.R.S.T. trials and third–party materials; no data were generated here. The scale difference in soybean meal values between soybean variety rankings – Illinois [4] and the present study likely reflects the variation in soybean nutrient composition, price differences of ingredients used in feed formulation, and the method of calculation (feed formulation software vs. Excel-based calculations). The LFV score facilitated the selection of soybean varieties with higher feed value, a trait similar to disease or drought resistance, to improve meal value and nutrient composition. Soybean LFV scores of the 47 varieties in the present study were calculated based on the value of SBM in swine (nursery, finisher, and lactation) and poultry (broiler and layer) diets [7,11]. Higher CP and amino acid concentrations in soybean varieties led to higher SBM values and, in turn, higher LFV scores. This is reflected in the LFV scores of soybean varieties in the present study, with varieties having lower LFV scores (1.0–4.0) showing lower soybean yield, CP, and amino acid concentrations than varieties with high LFV scores.

### 4.3. Relative Soybean Meal Values for Livestock

Relative soybean meal value was calculated following the general procedure employed by the HY + Q program prior to the integration of Dalex software [4]. Thereafter, the HY + Q program utilized an automated, multi-step least-cost formulation model developed by Dalex Livestock Solutions to estimate the economic value of large numbers of soybean meal samples. Because the HY + Q program has been discontinued, detailed information on its formulation procedures, ingredient prices, and computational methods is no longer available, preventing exact replication of the original software-driven methodology. Thus, the present study employed a simplified Excel-based approach to estimate soybean meal value. Differences in soybean meal composition, ingredient prices, and formulation assumptions may consequently influence the estimated soybean meal values. As the price-sensitivity analysis was not carried out for the least-cost feed formulation, the relative soybean meal value and the ranking of soybeans should be interpreted as scenario-specific, and not as predictions.

Overall, the LFV scores of soybean varieties in the present study that required <34.5% inclusion in the diets averaged 7.4 ± 0.51, while the LFV scores of soybean varieties that required >36.0% inclusion in the diet averaged 3.5 ± 1.72. Similarly, the CP concentration of soybean varieties that required >36.0% inclusion in the diet averaged 38.1 ± 0.49% while that which required <34.5% inclusion in the diets averaged 42.3 ± 0.35%. These results suggest that soybean varieties with greater CP and amino acid concentrations and higher relative soybean meal value and relative livestock feed value scores may require lower inclusion rates in modeled backgrounding beef cattle diets to meet metabolizable protein requirements. It should be noted that no actual feeding trial using growing beef cattle was carried out for this research. The protein and amino acid concentrations of soybean samples evaluated in the present study were incorporated into model backgrounding beef cattle diets to evaluate nutrient composition and N and CH_4_ emissions (Supplementary Files S1 and S2). Greater dietary CP was associated with an increase in total N intake and urinary and fecal N excretion. It should also be noted that when modeled SBM was included in the diets to have a positive metabolizable protein balance, the resulting dietary CP concentrations (22.4–23.5%) were higher than those required for typical backgrounding beef cattle (12–14%). Although the modeled diets supplied MP near the predicted requirement, they also supplied RDP in excess of the modeled requirement. Therefore, the relatively high dietary CP concentrations reflect the standardized ingredient and formulation constraints used for the comparative simulations rather than the biological CP requirement of growing cattle. These diets should consequently be considered comparative model scenarios rather than protein-optimized practical formulations. Because rumen fermentation is the primary source of GHG emissions in ruminants relative to non-ruminant animals, it is apparent that lower dietary CP inclusion, and possibly the use of lower-quality SBM, may be sufficient to meet the CP requirements of beef cattle. Moreover, urinary and fecal N excretion could be reduced by lowering CP concentrations in the diets of backgrounding beef cattle. These predicted observations from modeled beef cattle diets should be verified by feeding trials using beef cattle before finalizing the biological significance of the present study’s observations. In an evaluation of over 8000 soybean seed samples, Mourtzinis et al. [26] reported that there is a strong relationship between CP concentration and the value of SBM. The yield of soybean varieties that required <34.5% inclusion in the diets averaged 55.5 ± 3.16 bu/ac (3732.4 kg/ha), while that of soybean varieties that required >36.0% inclusion in the diet was 39.6 ± 6.93 bu/ac (2663.1 kg/ha) in the present study. In contrast, Mourtzinis et al. [26] reported no relationship between soybean seed yield and SBM value, indicating that selecting soybeans with higher SBM value among high-yielding varieties can yield the greatest returns for soybean producers.

Soybean seed varieties with higher protein concentrations were associated with a lower SBM inclusion in diets (kg/ton of feed) while increasing dietary CP concentrations. Greater dietary N concentrations increase urinary and fecal N excretion [38] and decreases N use effieiency [39]). The model predicted greater nitrogen retention, and potentially improved nitrogen-use efficiency, in diets with lower crude protein concentrations. These lower concentrations were achieved with soybean meal of relatively lower meal value. SBM with higher feed value likely commands a premium price despite its lower dietary inclusion, potentially encouraging adoption of soybean cultivars with superior meal quality. However, higher soybean seed protein concentrations in the modeled beef cattle diets were associated with predicted greater N intake, greater fecal and urinary N losses, and lower N retention, indicating that ruminants may derive limited benefit from the superior-quality SBM required by the non-ruminant species such as swine and poultry. Notably, protein quality is less critical for ruminants than for non-ruminants because of extensive protein degradation and ruminants’ ability to use non-protein nitrogen sources such as urea to produce microbial CP in the rumen. Further dietary evaluation of soybean seed protein concentrations across dietary inclusion levels and livestock emissions from ruminant and non-ruminant species will provide detailed information on the biological role of SBM quality in the growth performance of livestock species.

The observed increase in CH_4_ emission metrics associated with increasing dietary CP concentration should be interpreted with caution, as the relative range for CH_4_ production was comparatively small (approximately 1.8%) and within the model’s resolution and of limited practical significance. Mechanistically, CH_4_ production is generally driven by carbohydrate fermentation rather than CP concentration. The CH_4_ production metrics were model outputs when modeled SBM was incorporated into background beef cattle diets. Because the percent of SBM and feed corn inclusion was fixed, any change in SBM inclusion was associated with a proportionate change in feed corn inclusion in the diets. SBM with greater LFV scores was needed at lower inclusion in the diets, which subsequently increased the level of feed corn inclusion and ultimately impacted the predicted CH_4_ production metrics.

Conventionally, soybean producers select varieties with the highest yield, as soybean compositional quality is often secondary. Soybeans are conventionally priced based on protein and oil content. However, the convention of a minimum protein content of >35% and an oil content of >19% often failed to capture the highest prices for soybean producers. Mourtzinis et al. [26] reported that using only the minimum protein and oil content as the SBM indicator often fails to explain variability in soybean meal value, whereas including essential amino acid concentrations helps to accurately estimate it. The value of soybean meal can vary significantly among soybean varieties marketed by commercial seed suppliers, suggesting that soybean producers can identify high-yielding varieties with higher soybean meal value.

### 4.4. Limitations of This Study

It should be noted that in the present study, soybean varieties were sampled from commercial production fields and variety trial plots, limiting the opportunities for direct comparison between varieties over locations and crop years. Further, the majority of the samples were not equally represented in both years of the study. Soybean varieties from seed supplier 1 were evaluated only at Carbondale in 2022; seed supplier 3 only at Ewing in both 2022 and 2023; and seed suppliers 4 and 5 only at Ewing in 2022. The effects of crop year, location, and seed supplier should be interpreted with caution because soybean varieties were not equally represented across years, locations, and seed suppliers. The unbalanced sample distribution may have confounded the estimated effects, limiting the ability to distinguish true year, location, or supplier effects from differences associated with unequal variety representation. Furthermore, in the absence of a price-sensitivity analysis of the least-cost feed formulation used to evaluate soybean varieties, the relative soybean meal values and rankings of soybean varieties should be interpreted with caution. These results represent scenario-specific estimates based on the ingredient prices used in the present analysis rather than predictions applicable across varying market conditions.

However, the intent of the research was not to directly compare soybean varieties or commercial soybean suppliers, but to assess the relative variability in nutrient composition and meal value among commercially available soybean varieties in southern Illinois and their impact on livestock nutrition. Further evaluation of soybean varieties sampled over multiple crop years for the nutrient composition, soybean meal values, and LFV scores, and the impact on inclusion of the meal in livestock diets (non-ruminant and ruminant diets) will provide detailed information on dietary inclusion requirements, demand for feed corn and other ingredients, and livestock emissions for potential adoption of soybean varieties with greater LFV scores to capture highest prices for soybean producers.

## 5. Conclusions

This study’s results indicated that soybean varieties vary considerably in CP and amino acid concentrations, leading to variation in soybean meal values and LFV scores. Soybean varieties with higher LFV scores and soybean meal values were associated with lower SBM inclusion and greater feed corn inclusion in modeled beef cattle diets. These results suggest that improved soybean meal quality may increase the use of feed corn in these diets. The negative association between relative soybean meal value and SBM inclusion, SBM inclusion and TMR CP, and TMR CP and retained N in the modeled backgrounding beef cattle diets likely indicates that backgrounding beef cattle may not require the superior quality SBM preferred for non-ruminant species. Under the single-price scenario evaluated in the present study, differences in soybean meal value may have implications for its marketing and use across livestock classes. Selecting high-yielding soybean varieties with relatively higher soybean meal value may offer an opportunity to improve returns for soybean producers. Large-scale field studies conducted across diverse geographic locations and multiple crop years could provide a more comprehensive understanding of varietal effects on LFV scores and soybean meal value. Complementary livestock feeding trials could also help validate predicted nutrient utilization, N balance, and CH_4_ production.

## Figures and Tables

**Table 1:** List of soybean samples collected from Carbondale and Ewing locations during the 2022–2023 crop years.

	Carbondale		Ewing	
Seed Supplier	2022	2023	2022	2023
1	7	-	-	-
2	-	2	-	4
3	-	-	10	10
4	-	-	8	-
5	-	-	6	-

Note: Only seed supplier 3 had soybean varieties in both 2022 and 2023 at Ewing.

**Table 2:** Agronomic characteristics and nutrient composition of soybean samples collected from Carbondale and Ewing locations during the 2022 and 2023 crop years.

Seed Source	Location	Crop Year	Relative Maturity	Technology	SUR	SCNR	Yield, bu/ac	Seed DM	Seed CP
1	Carbondale	2022	3.3	E3	No	Good	57.0	90.2	38.8
1	Carbondale	2022	3.6	E3	Yes	Good	58.0	89.9	39.4
1	Carbondale	2022	3.7	E3	Yes	Moderate	.	89.4	38.5
1	Carbondale	2022	4.0	E3	No	Good	51.0	90.4	40.3
1	Carbondale	2022	4.5	E3	No	Good	47.0	90.4	40.0
1	Carbondale	2022	4.6	E3	Yes	Good	44.0	90.1	40.1
1	Carbondale	2022	4.9	E3	Yes	Good	41.0	90.3	39.8
3	Ewing	2022	3.7	E3	No	Moderate	46.6	93.5	40.7
3	Ewing	2022	4.0	E3	No	NA	48.9	93.2	40.1
3	Ewing	2022	4.4	E3	No	NA	46.8	93.0	41.1
3	Ewing	2022	4.2	E3	No	Good	50.7	93.0	40.3
3	Ewing	2022	4.6	E3	No	Good	47.6	92.9	39.2
3	Ewing	2022	3.5	E3	No	Good	47.2	92.9	39.9
3	Ewing	2022	4.8	E3	No	Good	50.1	92.2	38.2
3	Ewing	2022	4.5	E3	No	Good	46.7	92.8	39.1
3	Ewing	2022	3.8	E3	No	NA	47.3	93.4	40.6
3	Ewing	2022	4.0	E3	No	Good	46.8	93.2	40.4
4	Ewing	2022	3.3	RXF	No	Moderate	54.2	93.1	42.0
4	Ewing	2022	3.5	RXF	No	Good	58.1	93.2	41.8
4	Ewing	2022	3.6	RXF	No	Good	55.5	93.1	41.2
4	Ewing	2022	4.0	RXF	Yes	Moderate	52.8	93.1	41.2
4	Ewing	2022	4.2	RXF	Yes	Good	59.1	92.9	42.2
4	Ewing	2022	4.5	RXF	Yes	Good	.	92.7	42.9
4	Ewing	2022	4.7	RXF	Yes	Good	59.3	92.7	40.5
4	Ewing	2022	4.8	RXF	Yes	Good	53.2	92.7	42.3
5	Ewing	2022	3.4	E3	Yes	Good	50.2	93.0	41.3
5	Ewing	2022	3.6	E3	Yes	Good	57.2	93.1	41.1
5	Ewing	2022	3.9	E3	No	Good	47.7	92.9	41.5
5	Ewing	2022	4.1	E3	No	Good	51.6	93.0	39.6
5	Ewing	2022	4.4	E3	Yes	Good	52.8	92.8	40.9
5	Ewing	2022	4.8	E3	No	Good	49.8	92.7	40.4
2	Carbondale	2023	4.2	RXF	Yes	Good	.	90.7	40.2
2	Carbondale	2023	4.8	RXF	Yes	Good	41.0	90.5	39.2
3	Ewing	2023	3.1	E3	Yes	Good	35.1	91.2	40.0
3	Ewing	2023	3.3	E3	Yes	Good	42.0	89.6	40.8
3	Ewing	2023	3.4	E3	Yes	Good	35.6	91.5	39.4
3	Ewing	2023	3.8	E3	No	Moderate	41.8	89.6	38.6
3	Ewing	2023	4.0	E3	No	Good	38.2	89.2	40.6
3	Ewing	2023	4.2	E3	No	Good	39.6	90.1	39.2
3	Ewing	2023	4.4	E3	No	Good	39.3	90.7	38.5
3	Ewing	2023	4.5	E3	No	Good	29.1	91.2	37.6
3	Ewing	2023	4.6	E3	No	Good	31.4	89.7	40.2
3	Ewing	2023	4.8	E3	No	Good	.	89.7	42.1
2	Ewing	2023	3.0	E3	No	Good	36.1	91.5	40.2
2	Ewing	2023	3.4	E3	Yes	Good	.	91.7	37.7
2	Ewing	2023	4.4	E3	Yes	Good	36.1	91.3	38.3
2	Ewing	2023	3.9	E3	No	Good	41.2	91.3	37.3

Note: Technology E3 indicates three herbicide tolerances—2,4-D choline, glyphosate, and glufosinate; RXF denotes Roundup Ready 2 XtendFlex that provides resistance to dicamba, glyphosate, and glufosinate; SUR, sulfonylurea tolerant; SCNR, soybean cyst nematode resistance; NA, not available; “.” Indicates missing values.

**Table 3:** Effects of soybean relative maturity groups on the nutrient composition and meal value of soybeans over two crop years.

	Soybean Relative Maturity Groups			
Item	3.0–3.5	3.6–4.0	4.1–4.5	4.6–5.0
Number of samples	10	14	13	10
DM, %	91.8 ± 1.26	91.8 ± 1.73	91.9 ± 1.16	91.4 ± 1.39
Yield, bushels/ac (kg/ha) ^1^	46.2 ± 9.32 (3104.8)	48.7 ± 6.14 (3274.6)	45.3 ± 8.59 (3049.5)	46.4 ± 8.13 (3119.0)
CP, %	40.2 ± 1.35	40.1 ± 1.23	40.0 ± 1.53	40.2 ± 1.26
Relative soybean meal values, US$/ton	356.3 ± 4.44	356.5 ± 5.76	354.7 ± 7.77	355.4 ± 4.24
Amino acid concentrations, % DM basis				
Cysteine	0.69 ± 0.02	0.69 ± 0.05	0.69 ± 0.06	0.67 ± 0.05
Isoleucine	2.07 ± 0.11	2.07 ± 0.08	2.08 ± 0.09	2.07 ± 0.06
Lysine	2.89 ± 0.12	2.88 ± 0.09	2.88 ± 0.10	2.86 ± 0.05
Methionine	0.60 ± 0.02	0.59 ± 0.02	0.59 ± 0.02	0.59 ± 0.02
Threonine	1.66 ± 0.06	1.65 ± 0.05	1.65 ± 0.05	1.65 ± 0.04
Tryptophan	0.40 ± 0.04	0.41 ± 0.03	0.39 ± 0.04	0.41 ± 0.04
Valine	2.11 ± 0.11	2.11 ± 0.06	2.12 ± 0.09	2.12 ± 0.06

Note: Relative soybean meal values were calculated by converting the seed composition to modeled SBM composition, assuming 12% moisture and 74% meal yield, and using the SBM use (kg) in swine and poultry diets, total diet costs (US$/ton), and current market ingredient prices (US$/ton). ^1^ Yield data of two samples from 2022 and three samples from 2023 were missing. Observations lacking yield data were excluded only from analysis involving yield. Amino acid composition was analyzed at the Agricultural Experiment Station Chemical Laboratories, Columbia, MO, USA.

**Table 4:** Soybean seeds collected from Carbondale and Ewing locations over the 2022 and 2023 crop years, with relative soybean meal values for various classes of livestock.

			Relative Soybean Meal Values for Livestock Species					
Seed Source	Location	Crop Year	Swine–Nursery	Swine–Finishing	Swine–Lactation	Broiler	Layer	Average
1	Carbondale	2022	351.6	349.5	350.7	348.3	349.0	349.8
1	Carbondale	2022	354.3	352.7	359.9	359.1	358.1	356.8
1	Carbondale	2022	353.6	352.5	355.7	352.6	352.2	353.3
1	Carbondale	2022	356.9	355.7	357.7	356.8	355.4	356.5
1	Carbondale	2022	350.7	349.9	353.7	352.2	350.0	351.3
1	Carbondale	2022	357.6	356.2	363.6	361.2	360.3	359.8
1	Carbondale	2022	353.6	357.4	360.2	358.8	357.2	357.4
3	Ewing	2022	364.2	363.2	367.6	366.2	367.3	365.7
3	Ewing	2022	353.7	356.7	357.0	358.2	356.5	356.4
3	Ewing	2022	365.5	364.6	368.1	370.0	368.3	367.3
3	Ewing	2022	359.7	359.0	361.0	362.2	362.4	360.8
3	Ewing	2022	350.6	349.0	350.0	349.7	349.0	349.7
3	Ewing	2022	357.4	356.6	360.5	355.9	356.3	357.3
3	Ewing	2022	352.7	351.3	352.4	351.9	351.2	351.9
3	Ewing	2022	358.5	357.3	357.5	358.2	357.2	357.7
3	Ewing	2022	355.9	354.0	362.7	357.5	360.2	358.1
3	Ewing	2022	362.3	360.5	368.0	365.3	365.0	364.2
4	Ewing	2022	354.9	356.3	362.4	358.7	357.0	357.9
4	Ewing	2022	359.7	357.4	367.6	361.4	362.9	361.8
4	Ewing	2022	359.2	357.4	364.3	362.1	362.2	361.0
4	Ewing	2022	355.8	354.9	358.2	356.6	354.9	356.1
4	Ewing	2022	358.8	359.6	366.6	360.4	355.1	360.1
4	Ewing	2022	357.4	355.0	368.2	359.8	359.8	360.1
4	Ewing	2022	353.4	353.5	359.0	353.5	353.8	354.7
4	Ewing	2022	354.7	352.0	365.4	360.5	359.5	358.4
5	Ewing	2022	360.9	358.9	368.9	362.9	364.3	363.2
5	Ewing	2022	356.2	355.2	358.9	355.9	355.4	356.3
5	Ewing	2022	355.0	354.7	359.6	355.6	354.7	355.9
5	Ewing	2022	358.3	356.8	359.7	357.7	356.8	357.8
5	Ewing	2022	357.1	355.2	364.2	361.3	359.9	359.5
5	Ewing	2022	348.4	348.8	354.5	350.1	348.9	350.2
3	Ewing	2023	357.4	355.6	356.7	355.8	355.2	356.1
3	Ewing	2023	356.8	357.8	364.8	358.3	357.9	359.1
3	Ewing	2023	352.6	351.4	353.8	351.7	351.5	352.2
3	Ewing	2023	349.3	350.5	356.4	349.0	350.4	351.1
3	Ewing	2023	353.5	358.1	365.1	356.5	357.9	358.2
3	Ewing	2023	350.8	350.0	354.0	350.6	350.2	351.1
3	Ewing	2023	340.0	340.6	346.3	340.6	340.7	341.6
3	Ewing	2023	340.5	340.1	344.2	340.1	340.2	341.0
3	Ewing	2023	356.4	357.0	363.1	356.3	356.8	357.9
3	Ewing	2023	358.9	356.1	368.4	363.9	361.8	361.8
2	Ewing	2023	352.9	350.7	350.5	352.1	350.4	351.3
2	Ewing	2023	353.5	346.3	357.6	356.7	356.9	354.2
2	Ewing	2023	348.4	347.3	349.7	347.1	347.6	348.0
2	Ewing	2023	342.1	340.9	343.3	340.8	341.0	341.6
2	Carbondale	2023	352.8	353.6	360.6	354.4	353.9	355.0
2	Carbondale	2023	352.4	351.1	354.2	352.1	351.8	352.3

Note: Relative soybean meal values were calculated by converting the seed composition to modeled SBM composition, assuming 12% moisture and 74% meal yield, and using the SBM use (kg) in swine and poultry diets, total diet costs (US$/ton), and current market ingredient prices (US$/ton).

**Table 5:** Effects of nutrient composition of soybean varieties on relative soybean meal values (US$/ton).

	Relative Soybean Meal Value Category				
	Low	Medium	High		
Item	≤US$350/ton	US$350.1–US$360/ton	≥US$360.1/ton	SEM	*p*-Value
Number of samples	6	31	10	-	-
Relative maturity	3.3–4.6	3.0–4.9	3.4–4.8	-	-
DM, %	91.3 ^b^	91.5 ^ab^	92.7 ^a^	0.53	0.03
Yield, bushels/ac (kg/ha) ^1^	41.7 (2805.5) ^b^	46.5 (3123.8) ^ab^	51.7 (3478.6) ^a^	3.02 (203.2)	0.05
CP, % DM	38.3 ^c^	40.1 ^b^	41.4 ^a^	0.40	<0.0001
Relative soybean meal values, US$/ton	345.3 ^c^	355.5 ^b^	362.6 ^a^	1.22	<0.0001
Amino acid concentrations, % DM basis					
Lysine	2.74 ^c^	2.87 ^b^	2.99 ^a^	0.023	<0.0001
Methionine	0.57 ^c^	0.59 ^b^	0.62 ^a^	0.007	<0.0001
Cysteine	0.66 ^b^	0.68 ^b^	0.73 ^a^	0.018	<0.01
Threonine	1.58 ^c^	1.65 ^b^	1.71 ^a^	0.011	<0.0001
Tryptophan	0.37 ^b^	0.40 ^ab^	0.42 ^a^	0.014	0.02
Isoleucine	1.97 ^c^	2.06 ^b^	2.16 ^a^	0.027	<0.001
Valine	2.00 ^c^	2.11 ^b^	2.19 ^a^	0.026	<0.0001

Note: Relative soybean meal values were calculated by converting the seed composition to modeled SBM composition, assuming 12% moisture and 74% meal yield, and using the SBM use (kg) in swine and poultry diets, total diet costs (US$/ton), and current market ingredient prices (US$/ton). Relative soybean meal values ranged from the base value of US$340/ton to US$370/ton for the various livestock classes (swine and poultry) and were categorized into low (≤US$350/ton, medium (US$350.1 to US$360/ton), and high (≥US$360.1/ton). ^1^ Yield data of two samples from 2022 and three samples from 2023 were missing. Observations lacking yield data were excluded only from analysis involving yield. Amino acid composition was analyzed at the Agricultural Experiment Station Chemical Laboratories Columbia, MO, USA. ^a–c^ within a row, means without a common letter differ (*p* < 0.05) among relative soybean meal value categories.

**Table 6:** Soybean seeds collected from Carbondale and Ewing locations over the 2022 and 2023 crop years with relative livestock feed value (LFV) scores.

			Relative Livestock Feed Value Scores (LFV) for Swine and Poultry					
Seed Source	Location	Crop Year	Swine–Nursery	Swine–Finishing	Swine–Lactation	Broiler	Layer	Average
1	Carbondale	2022	5.0	4.4	4.3	3.4	3.8	4.2
1	Carbondale	2022	6.0	5.6	7.2	6.7	6.7	6.4
1	Carbondale	2022	5.8	5.5	5.8	4.7	4.8	5.3
1	Carbondale	2022	6.9	6.7	6.5	6.0	5.8	6.4
1	Carbondale	2022	4.7	4.6	5.2	4.6	4.1	4.6
1	Carbondale	2022	7.2	6.9	8.3	7.3	7.4	7.4
1	Carbondale	2022	5.7	7.3	7.2	6.6	6.4	6.6
3	Ewing	2022	9.5	9.5	9.5	8.8	9.6	9.4
3	Ewing	2022	5.8	7.1	6.2	6.4	6.2	6.3
3	Ewing	2022	10.0	10.0	9.7	10.0	10.0	9.9
3	Ewing	2022	7.9	7.9	7.5	7.6	8.1	7.8
3	Ewing	2022	4.7	4.3	4.1	3.9	3.8	4.2
3	Ewing	2022	7.1	7.0	7.3	5.7	6.1	6.6
3	Ewing	2022	5.4	5.1	4.8	4.5	4.5	4.9
3	Ewing	2022	7.5	7.3	6.4	6.4	6.4	6.8
3	Ewing	2022	6.6	6.1	8.0	6.2	7.4	6.9
3	Ewing	2022	8.8	8.5	9.7	8.6	8.9	8.9
4	Ewing	2022	6.2	6.9	7.9	6.6	6.3	6.8
4	Ewing	2022	7.9	7.3	9.5	7.4	8.2	8.1
4	Ewing	2022	7.7	7.3	8.5	7.6	8.0	7.8
4	Ewing	2022	6.5	6.4	6.6	5.9	5.7	6.2
4	Ewing	2022	7.6	8.1	9.2	7.1	5.7	7.5
4	Ewing	2022	7.1	6.5	9.7	6.9	7.3	7.5
4	Ewing	2022	5.7	5.9	6.9	5.0	5.3	5.8
4	Ewing	2022	6.1	5.4	8.9	7.1	7.2	6.9
5	Ewing	2022	8.3	7.9	10.0	7.8	8.7	8.5
5	Ewing	2022	6.7	6.5	6.8	5.7	5.9	6.3
5	Ewing	2022	6.3	6.1	7.0	5.6	5.6	6.1
5	Ewing	2022	7.4	7.1	7.1	6.3	6.3	6.8
5	Ewing	2022	7.0	6.5	8.5	7.3	7.3	7.3
5	Ewing	2022	3.9	4.2	5.5	4.0	3.8	4.3
3	Ewing	2023	7.1	6.7	6.2	5.7	5.8	6.3
3	Ewing	2023	6.9	7.5	8.7	6.5	6.6	7.2
3	Ewing	2023	5.4	5.1	5.2	4.5	4.6	5.0
3	Ewing	2023	4.2	4.8	6.0	3.7	4.3	4.6
3	Ewing	2023	5.7	7.6	8.7	5.9	6.7	6.9
3	Ewing	2023	4.7	4.6	5.3	4.1	4.2	4.6
3	Ewing	2023	1.0	1.2	2.9	1.1	1.2	1.5
3	Ewing	2023	1.1	1.0	1.2	1.0	1.0	1.3
3	Ewing	2023	6.7	7.2	8.1	5.9	6.3	6.8
3	Ewing	2023	7.6	6.9	9.8	8.1	7.9	8.1
2	Ewing	2023	5.5	4.9	4.2	4.6	4.3	4.7
2	Ewing	2023	5.7	3.3	6.4	6.0	6.3	5.5
2	Ewing	2023	3.9	3.6	4.0	3.1	3.4	3.6
2	Ewing	2023	1.7	1.3	2.0	1.2	1.3	1.5
2	Carbondale	2023	5.5	5.9	7.3	5.3	5.4	5.9
2	Carbondale	2023	5.3	5.0	5.4	4.6	4.7	5.0

Note: Relative livestock feed value scores of soybean varieties were calculated using the relative soybean meal value, with the variety requiring the smallest quantity in the diet formulation to meet the amino acid, nutrient, and energy requirements of the livestock species receiving the highest relative livestock feed value score.

**Table 7:** Effects of nutrient composition on the relative livestock feed value (LFV) scores of soybean varieties.

	Relative Livestock Feed Value (LFV) Scores of Soybean Varieties				
Item	Low (1.0–4.0)	Medium (4.1–7.0)	High (7.1–10.0)	SEM	*p*-Value
Number of samples	4	30	13	-	-
Relative maturity	3.9–4.5	3.0–4.9	3.3–4.8	-	-
DM, %	91.1	91.6	92.3	0.68	0.19
Yield, bushels/ac (kg/ha) ^1^	36.4 (2449.6) ^b^	46.9 (3154.3) ^a^	50.2 (3378.5) ^a^	3.52 (91.0)	<0.01
CP, %	37.9 ^c^	39.9 ^b^	41.2 ^a^	0.49	<0.0001
Relative soybean meal value, US$/ton	343.1 ^c^	354.8 ^b^	361.9 ^a^	1.43	<0.0001
Amino acid concentrations, % DM basis					
Lysine	2.73 ^c^	2.86 ^b^	2.97 ^a^	0.032	<0.0001
Methionine	0.57 ^c^	0.59 ^b^	0.61 ^a^	0.009	<0.001
Cysteine	0.64 ^b^	0.68 ^b^	0.72 ^a^	0.022	<0.01
Threonine	1.57 ^c^	1.64 ^b^	1.70 ^a^	0.017	<0.0001
Tryptophan	0.35 ^b^	0.40 ^a^	0.42 ^a^	0.016	<0.01
Isoleucine	1.98 ^b^	2.05 ^b^	2.15 ^a^	0.034	<0.0001
Valine	2.02 ^b^	2.10 ^b^	2.18 ^a^	0.033	<0.0001

Note: Relative livestock feed value scores of soybean varieties were calculated using the relative soybean meal value with the variety requiring the smallest quantity in the diet formulation to meet the amino acids, nutrient, and energy requirements of the livestock species receiving the highest relative livestock feed value score. Relative soybean meal values were calculated by converting the seed composition to modeled SBM composition, assuming 12% moisture and 74% meal yield, and using the SBM use (kg) in swine and poultry diets, total diet costs (US$/ton), and current market ingredient prices (US$/ton). ^1^ Yield data of two samples from 2022 and three samples from 2023 were missing. Observations lacking yield data were excluded only from analysis involving yield. Amino acid composition was analyzed at the Agricultural Experiment Station Chemical Laboratories Columbia, MO, USA. ^a–c^ within a row, means without a common letter differ (*p* < 0.05) among relative soybean meal value categories.

**Table 8:** Maximum, minimum, and mean temperatures (mean ± SD) and cumulative precipitation during soybean production at Carbondale and Ewing (IL) during 2022 and 2023 crop years.

	Carbondale		Ewing	
Items	2022	2023	2022	2023
Maximum temperature, °C (°F)	28.3 ± 3.82 (83.0 ± 6.88)	27.8 ± 3.70 (82.1 ± 6.66)	27.9 ± 4.39 (82.3 ± 7.91)	27.3 ± 3.98 (81.1 ± 7.17)
Minimum temperature, °C (°F)	14.7 ± 5.51 (58.5 ± 9.92)	14.8 ± 4.46 (58.6 ± 8.04)	16.5 ± 6.15 (61.7 ± 11.1)	15.6 ± 4.18 (60.0 ± 7.52)
Mean temperature, °C (°F)	21.4 ± 4.67 (70.6 ± 8.41)	21.1 ± 3.99 (70.1 ± 7.17)	20.8 ± 4.75 (69.5 ± 8.55)	21.2 ± 3.94 (70.2 ± 7.08)
Cumulative precipitation, mm (inches)	370.6 (14.6)	540.3 (21.3)	431.3 (17.0)	467.6 (18.4)

**Table 9:** Effects of crop year on the nutrient composition and relative soybean meal values across locations and seed sources.

	Crop Year			
Item	2022	2023	SEM	*p*-Value
Number of samples	31	16	-	-
Relative maturity	3.3–4.9	3.0–4.8	-	-
DM, %	91.7	89.8	0.19	<0.0001
Yield, bushels/ac (kg/ha) ^1^	51.0 (3426.7)	37.1 (2496.0)	1.29 (86.7)	<0.0001
CP, % DM	40.3	39.1	0.30	<0.01
Relative soybean meal value, US$/ton	357.2	351.2	1.29	<0.001
Amino acid concentrations, % DM basis				
Cysteine	0.70	0.65	0.011	<0.001
Isoleucine	2.08	2.01	0.020	0.01
Lysine	2.89	2.79	0.020	<0.001
Methionine	0.60	0.58	0.005	0.06
Threonine	1.66	1.61	0.011	<0.001
Tryptophan	0.41	0.40	0.009	0.56
Valine	2.12	2.07	0.020	0.06

Note: Relative soybean meal values were calculated by converting the seed composition to modeled SBM composition, assuming 12% moisture and 74% meal yield, and using the SBM use (kg) in swine and poultry diets, total diet costs (US$/ton), and current market ingredient prices (US$/ton). ^1^ Yield data of two samples from 2022 and three samples from 2023 were missing. Observations lacking yield data were excluded only from analysis involving yield. Amino acid composition was analyzed at the Agricultural Experiment Station Chemical Laboratories Columbia, MO, USA.

**Table 10:** Effects of location on the nutrient composition and relative soybean meal values across years and seed sources.

	Location			
Item	Carbondale	Ewing	SEM	*p*-Value
Number of samples	9	38	-	-
Relative maturity	3.3–4.9	3.0–4.8	-	-
DM, %	89.7	91.8	0.20	<0.0001
Yield, bushels/ac (kg/ha) ^1^	43.9 (2950.4)	44.2 (2972.3)	1.41 (94.8)	0.87
CP, % DM	39.3	40.1	0.32	0.07
Relative soybean meal value, US$/ton	353.2	355.2	1.40	0.32
Amino acid concentrations, % DM basis				
Cysteine	0.67	0.68	0.012	0.85
Isoleucine	2.02	2.07	0.022	0.08
Lysine	2.81	2.88	0.022	0.02
Methionine	0.59	0.59	0.006	0.44
Threonine	1.63	1.65	0.012	0.21
Tryptophan	0.41	0.40	0.010	0.50
Valine	2.08	2.12	0.022	0.24

Note: Relative soybean meal values were calculated by converting the seed composition to modeled SBM composition, assuming 12% moisture and 74% meal yield, and using the SBM use (kg) in swine and poultry diets, total diet costs (US$/ton), and current market ingredient prices (US$/ton). ^1^ Yield data of two samples from 2022 and three samples from 2023 were missing. Observations lacking yield data were excluded only from analysis involving yield. Amino acid composition was analyzed at the Agricultural Experiment Station Chemical Laboratories Columbia, MO, USA.

**Table 11:** Effects of seed supplier on the nutrient composition and relative soybean meal values over two crop years.

	Soybean Seed Supplier				
Item	1	2	3	4	5
Number of varieties	7	6	20	8	6
Year(s) sampled	2022	2023	2022, 2023	2022	2022
Location(s) sampled	Carbondale	Carbondale, Ewing	Ewing	Ewing	Ewing
Relative maturity	3.3–4.9	3.0–4.8	3.1–4.8	3.3–4.8	3.4–4.8
DM, %	90.1 ± 0.36	91.2 ± 0.47	91.6 ± 1.55	93 ± 0.21	92.9 ± 0.15
Yield, bushels/ac ^1^	49.7 ± 6.92 (3340.1)	38.6 ± 2.89 (2595.9)	42.7 ± 6.46 (2869.9)	56.0 ± 2.78 (3768.0)	51.6 ± 3.26 (3466.8)
CP, % DM	39.6 ± 0.69	38.8 ± 1.25	39.8 ± 1.10	41.8 ± 0.76	40.8 ± 0.70
Relative soybean meal value, US$/ton ^1^	355.0 ± 3.60	350.4 ± 4.96	356.0 ± 6.97	358.8 ± 2.46	357.2 ± 4.32
Amino acid concentrations, % DM basis					
Cysteine	0.70 ± 0.024	0.68 ± 0.037	0.69 ± 0.067	0.69 ± 0.030	0.67 ± 0.038
Isoleucine	2.03 ± 0.0.6	2.00 ± 0.068	2.06 ± 0.071	2.17 ± 0.076	2.10 ± 0.072
Lysine	2.82 ± 0.063	2.81 ± 0.069	2.87 ± 0.091	2.98 ± 0.062	2.91 ± 0.077
Methionine	0.59 ± 0.018	0.60 ± 0.021	0.59 ± 0.024	0.60 ± 0.013	0.61 ± 0.027
Threonine	1.63 ± 0.040	1.62 ± 0.039	1.64 ± 0.053	1.69 ± 0.036	1.67 ± 0.046
Tryptophan	0.42 ± 0.029	0.37 ± 0.038	0.41 ± 0.036	0.39 ± 0.038	0.40 ± 0.024
Valine	2.08 ± 0.065	2.07 ± 0.083	2.10 ± 0.067	2.20 ± 0.078	2.14 ± 0.060

Note: Relative soybean meal values were calculated by converting the seed composition to modeled SBM composition, assuming 12% moisture and 74% meal yield, and using the SBM use (kg) in swine and poultry diets, total diet costs (US$/ton), and current market ingredient prices (US$/ton). ^1^ Yield data of two samples from 2022 and three samples from 2023 were missing. Observations lacking yield data were excluded only from analysis involving yield. Amino acid composition was analyzed at the Agricultural Experiment Station Chemical Laboratories Columbia, MO, USA.

**Table 12:** Composition and nutrient concentrations of diets for backgrounding beef cattle containing modeled SBM using the mechanistic model of BCNRM (2016) of the National Academies of Sciences, Engineering, and Medicine (NASEM, 2016).

Dietary Composition, % DM Basis	Inclusion Levels
Corn silage	40.0
Bromegrass hay	10.0
Corn grain + SBM	44.9
Vitamin–mineral supplement ^1^	5.1
Dietary ingredients, kg/ton	
Corn silage	400.0
Bromegrass hay	100.0
Corn grain	82.4–117.6
SBM	331.8–367.1
Vitamin–mineral supplement ^1^	50.6
Diet nutrient composition ^2^, % DM basis	
DM	55.0
CP	22.4–23.5
EE	4.78–4.94
NDF	31.2–31.6
Lignin	2.55–2.56
Starch	21.1–23.5
Ash	4.88–5.06
NEm, Mcal/kg	1.79
NEg, Mcal/kg	1.16

Note: Relative soybean meal values were calculated by converting the seed composition to modeled SBM composition, assuming 12% moisture and 74% meal yield, and using the SBM use (kg) in swine and poultry diets, total diet costs (US$/ton), and current market ingredient prices (US$/ton). ^1^ Supplement consisted of 4.6% salt, 5.5% Ca, 0.55% P, 1.8% Na, 0.41% Mg, 0.37% K, 0.18% S, and 5.3 mg Co, 203 mg Cu, 18.3 mg I, 182 mg Fe, 532 mg Mn, 1.5 mg Se, 602 mg Zn, 26 mg F and 430 mg monensin per kg and 44 100 IU vitamin A, 11 025 IU vitamin D and 550 IU vitamin E per kg supplement. ^2^ CP, crude protein; EE, ether extract; NDF, neutral detergent fiber; NEm and NEg, net energy for maintenance and gain, respectively.

**Table 13:** Descriptive Pearson correlation matrix of soybean seed protein concentration, soybean meal value, dietary composition, nitrogen balance, and CH_4_ emissions of backgrounding beef cattle.

Item ^1^	Seed CP	TMR CP	TMR Starch	Retained N	CH_4_, % GE	CH_4_, mol/d	CH_4_, Mcal/d	CH_4_, g/kg DM	Soybean Meal Value	SBM, kg
Seed CP	1.00	0.97	0.98	−0.37	0.94	0.98	0.89	0.08	0.72	−0.98
TMR CP		1.00	0.91	−0.50	0.85	0.91	0.81	0.08	0.74	−0.91
TMR Starch			1.00	−0.25	0.96	0.99	0.91	0.08	0.67	−1.00
Retained N				1.00	−0.23	−0.26	−0.17	−0.11	−0.37	0.25
CH_4_, % GE					1.00	0.97	0.93	0.09	0.63	−0.97
CH_4_, mol/d						1.00	0.91	0.08	0.68	−0.99
CH_4_, Mcal/d							1.00	0.18	0.58	−0.92
CH_4_, g/kg DM								1.00	0.04	−0.09
Soybean meal value									1.00	−0.68
SBM, kg										1.00

Note: Association involving variables that are mathematically derived from one another (such as TMR CP, retained N, TMR starch, etc.,) should not be interpreted as independent associations. Pearson correlation was calculated using pairwise complete observations; therefore, the number of observations could differ between variable pairs depending on data availability. ^1^ Dietary composition, nitrogen balance, and methane emission parameters were obtained from the BCNRM (2016) of the National Academies of Sciences, Engineering, and Medicine (2016) for growing beef cattle (350 kg). TMR CP, total mixed ration crude protein; N, nitrogen; CH_4_, methane; GE, gross energy; SBM kg, kg of SBM required in one ton of TMR; corn kg, kg of corn required in one ton of TMR. Soybean meal value is calculated using the seed composition to model SBM composition, assuming 12% moisture and 74% meal yield, and using the SBM use (kg) in swine and poultry diets, total diet costs (US$/ton), and current market ingredient prices (US$/ton).

## Data Availability

The raw data supporting the conclusions of this article will be made available by the authors on request.

## Supplementary Materials

The following supporting information can be downloaded at: https://www.mdpi.com/article/10.3390/ani16193105/s1, File S1: Dietary composition of formulated beef cattle diets containing modeled SBM from soybean samples collected from Carbondale and Ewing locations over the 2022 to 2023 crop years; Supplementary File S2: Nutrient composition, N balance, and CH4 emission metrics of formulated beef cattle diets containing modeled SBM from soybean samples collected from Carbondale and Ewing locations over the 2022 to 2023 crop years.

## Abbreviations

The following abbreviations are used in this manuscript:

AOAC	Association of Official Analytical Chemists
CP	Crude protein
DM	Dry matter
HY + Q	High yield plus quality
ISA	Illinois Soybean Association
LFV	Livestock feed value
SBM	Soybean meal
