# Supplementary material for: Southern Illinois Soybean Varieties: Implications for Relative Livestock Feed Value Scores, Modeled Soybean Meal Values, and Livestock Diet Formulation
Source: Animals (Basel). 2026 Oct 2;16(19):3105. doi: 10.3390/ani16193105 (PMC13663384; doi:10.3390/ani16193105)
Supplement: Supplementary file 1 [file animals-16-03105-s001.zip › animals-4445627-supplementary.pdf]

Supplementary File S1. Dietary composition of formulated beef cattle diets containing modeled SBM from soybean samples collected from Carbondale and Ewing locations over the 2022 to 2023 crop years

	Dietary composition, kg						Dietary requirement, kg/ton					
No	Brome hay	Corn silage	Corn grain	SBM	Supplement	Total	Brome hay	Corn silage	Corn grain	SBM	Supplement	Total
2	0.85	3.4	0.8	3.1	0.4	8.5	100.0	400.0	89.4	360.0	50.6	1000.0
3	0.85	3.4	0.8	3.0	0.4	8.5	100.0	400.0	92.9	356.5	50.6	1000.0
4	0.85	3.4	0.8	3.1	0.4	8.5	100.0	400.0	88.2	361.2	50.6	1000.0
5	0.85	3.4	0.8	3.0	0.4	8.5	100.0	400.0	96.5	352.9	50.6	1000.0
7	0.85	3.4	0.8	3.0	0.4	8.5	100.0	400.0	95.3	354.1	50.6	1000.0
8	0.85	3.4	0.8	3.0	0.4	8.5	100.0	400.0	96.5	352.9	50.6	1000.0
9	0.85	3.4	0.8	3.0	0.4	8.5	100.0	400.0	94.1	355.3	50.6	1000.0
10	0.85	3.4	0.8	3.0	0.4	8.5	100.0	400.0	98.8	350.6	50.6	1000.0
11	0.85	3.4	0.8	3.0	0.4	8.5	100.0	400.0	96.5	352.9	50.6	1000.0
12	0.85	3.4	0.9	3.0	0.4	8.5	100.0	400.0	100.0	349.4	50.6	1000.0
13	0.85	3.4	0.8	3.0	0.4	8.5	100.0	400.0	96.5	352.9	50.6	1000.0
14	0.85	3.4	0.8	3.0	0.4	8.5	100.0	400.0	91.8	357.6	50.6	1000.0
15	0.85	3.4	0.8	3.0	0.4	8.5	100.0	400.0	95.3	354.1	50.6	1000.0
16	0.85	3.4	0.7	3.1	0.4	8.5	100.0	400.0	87.1	362.4	50.6	1000.0
17	0.85	3.4	0.8	3.1	0.4	8.5	100.0	400.0	90.6	358.8	50.6	1000.0
18	0.85	3.4	0.8	3.0	0.4	8.5	100.0	400.0	98.8	350.6	50.6	1000.0
19	0.85	3.4	0.8	3.0	0.4	8.5	100.0	400.0	97.6	351.8	50.6	1000.0
20	0.85	3.4	0.9	2.9	0.4	8.5	100.0	400.0	107.1	342.4	50.6	1000.0
21	0.85	3.4	0.9	2.9	0.4	8.5	100.0	400.0	103.5	345.9	50.6	1000.0
22	0.85	3.4	0.9	3.0	0.4	8.5	100.0	400.0	101.2	348.2	50.6	1000.0
23	0.85	3.4	0.9	3.0	0.4	8.5	100.0	400.0	101.2	348.2	50.6	1000.0
24	0.85	3.4	0.9	2.9	0.4	8.5	100.0	400.0	108.2	341.2	50.6	1000.0
25	0.85	3.4	1.0	2.8	0.4	8.5	100.0	400.0	117.6	331.8	50.6	1000.0
26	0.85	3.4	0.8	3.0	0.4	8.5	100.0	400.0	97.6	351.8	50.6	1000.0
27	0.85	3.4	0.9	2.9	0.4	8.5	100.0	400.0	109.4	340.0	50.6	1000.0

28	0.85	3.4	0.9	3.0	0.4	8.5	100.0	400.0	101.2	348.2	50.6	1000.0
29	0.85	3.4	0.9	3.0	0.4	8.5	100.0	400.0	100.0	349.4	50.6	1000.0
30	0.85	3.4	0.9	3.0	0.4	8.5	100.0	400.0	102.4	347.1	50.6	1000.0
31	0.85	3.4	0.8	3.0	0.4	8.5	100.0	400.0	94.1	355.3	50.6	1000.0
32	0.85	3.4	0.9	3.0	0.4	8.5	100.0	400.0	100.0	349.4	50.6	1000.0
33	0.85	3.4	0.8	3.0	0.4	8.5	100.0	400.0	97.6	351.8	50.6	1000.0
34	0.85	3.4	0.8	3.0	0.4	8.5	100.0	400.0	95.3	354.1	50.6	1000.0
35	0.85	3.4	0.8	3.0	0.4	8.5	100.0	400.0	98.8	350.6	50.6	1000.0
36	0.85	3.4	0.8	3.0	0.4	8.5	100.0	400.0	92.9	356.5	50.6	1000.0
38	0.85	3.4	0.8	3.1	0.4	8.5	100.0	400.0	88.2	361.2	50.6	1000.0
39	0.85	3.4	0.8	3.0	0.4	8.5	100.0	400.0	97.6	351.8	50.6	1000.0
40	0.85	3.4	0.8	3.0	0.4	8.5	100.0	400.0	91.8	357.6	50.6	1000.0
41	0.85	3.4	0.8	3.1	0.4	8.5	100.0	400.0	88.2	361.2	50.6	1000.0
42	0.85	3.4	0.7	3.1	0.4	8.5	100.0	400.0	83.5	365.9	50.6	1000.0
43	0.85	3.4	0.8	3.0	0.4	8.5	100.0	400.0	96.5	352.9	50.6	1000.0
44	0.85	3.4	0.9	2.9	0.4	8.5	100.0	400.0	108.2	341.2	50.6	1000.0
45	0.85	3.4	0.8	3.0	0.4	8.5	100.0	400.0	96.5	352.9	50.6	1000.0
46	0.85	3.4	0.7	3.1	0.4	8.5	100.0	400.0	84.7	364.7	50.6	1000.0
47	0.85	3.4	0.7	3.1	0.4	8.5	100.0	400.0	87.1	362.4	50.6	1000.0
48	0.85	3.4	0.7	3.1	0.4	8.5	100.0	400.0	82.4	367.1	50.6	1000.0
49	0.85	3.4	0.8	3.0	0.4	8.5	100.0	400.0	96.5	352.9	50.6	1000.0
50	0.85	3.4	0.8	3.0	0.4	8.5	100.0	400.0	91.8	357.6	50.6	1000.0

Supplementary File S2. Nutrient composition, N balance, and CH_4 emission metrics of formulated beef cattle diets containing modeled SBM from soybean samples collected from Carbondale and Ewing locations over the 2022 to 2023 crop years

	Nutrient composition, % DM basis							N balance parameters and CH_4 metrics							
No	DM	CP	EE	NDF	Lignin	Starch	Ash	N intake, g/d	N retained, g/d	Urinary N, g/d	Fecal N, g/d	CH_4, % GE	CH_4, mol/d	CH_4, Mcal/d	CH_4, g/kg DM
2	55.01	22.76	4.9	31.4	2.6	21.6	5.0	309.6	38.8	175.3	95.5	4.29	7.77	1.65	14.63
3	55	22.89	4.9	31.4	2.6	21.8	5.0	311.3	38.8	176.9	95.7	4.29	7.79	1.66	14.66
4	55.01	22.7	4.9	31.4	2.6	21.5	5.0	308.7	38.8	174.5	95.4	4.28	7.77	1.65	14.62
5	54.99	23.15	4.9	31.4	2.6	22.0	5.0	314.8	38.7	180.0	96.1	4.30	7.81	1.66	14.69
7	55	23.09	4.9	31.4	2.6	22.0	5.0	314.0	38.7	179.3	96.0	4.30	7.80	1.66	14.68
8	54.99	23.07	4.9	31.4	2.6	22.0	5.0	313.7	38.8	179.0	96.0	4.30	7.80	1.66	14.68
9	55	23.04	4.9	31.4	2.6	21.9	5.0	313.3	38.7	178.6	95.9	4.30	7.80	1.66	14.68
10	54.99	23.23	4.9	31.3	2.6	22.2	5.0	316.0	38.7	181.0	96.2	4.30	7.81	1.66	14.70
11	54.99	23.08	4.9	31.4	2.6	22.0	5.0	313.9	38.8	179.2	96.0	4.30	7.80	1.66	14.69
12	54.99	23.34	4.9	31.3	2.6	22.3	5.0	317.5	38.7	182.3	96.4	4.30	7.82	1.66	14.72
13	54.99	23.13	4.9	31.4	2.6	22.0	5.0	314.6	38.7	179.8	96.1	4.30	7.80	1.66	14.69
14	55	22.85	4.9	31.4	2.6	21.7	5.0	310.7	38.8	176.3	95.6	4.29	7.78	1.66	14.65
15	55	23.01	4.9	31.4	2.6	22.0	5.0	312.9	38.8	178.3	95.9	4.30	7.80	1.66	14.68
16	55.01	22.62	4.9	31.4	2.6	21.4	5.0	307.6	38.8	173.6	95.3	4.28	7.76	1.65	14.61
17	55	22.85	4.9	31.4	2.6	21.6	5.0	310.8	38.7	176.4	95.7	4.29	7.78	1.66	14.65
18	54.99	23.2	4.9	31.3	2.6	22.2	5.0	315.5	38.7	180.7	96.2	4.30	7.81	1.66	14.70
19	54.99	23.15	4.9	31.3	2.6	22.1	5.0	314.8	38.8	180.0	96.1	4.30	7.81	1.66	14.69
20	54.98	23.44	4.8	31.3	2.6	22.7	4.9	318.8	38.8	183.6	96.4	4.31	7.84	1.67	14.76
21	54.98	23.5	4.9	31.3	2.6	22.5	5.0	319.6	38.7	184.2	96.6	4.31	7.83	1.67	14.74
22	54.99	23.35	4.9	31.3	2.6	22.4	5.0	317.5	38.7	182.4	96.4	4.30	7.82	1.66	14.72
23	54.99	23.32	4.9	31.3	2.6	22.4	5.0	317.2	38.7	182.1	96.3	4.30	7.82	1.66	14.72
24	54.97	23.44	4.8	31.2	2.6	22.8	4.9	318.8	38.8	183.7	96.4	4.32	7.85	1.67	14.77
25	54.96	23.33	4.8	31.2	2.6	23.5	4.9	317.3	38.8	182.5	96.0	4.34	7.89	1.68	14.85

26	54.99	23.17	4.9	31.3	2.6	22.1	5.0	315.1	38.7	180.2	96.1	4.30	7.81	1.66	14.70
27	54.97	23.45	4.8	31.2	2.6	22.9	4.9	318.9	38.8	183.7	96.4	4.32	7.86	1.67	14.79
28	54.99	23.37	4.9	31.3	2.6	22.4	5.0	317.8	38.7	182.6	96.4	4.30	7.82	1.66	14.72
29	54.99	23.34	4.9	31.3	2.6	22.3	5.0	317.4	38.7	182.3	96.4	4.30	7.82	1.66	14.72
30	54.98	23.42	4.9	31.3	2.6	22.4	5.0	318.5	38.7	183.3	96.5	4.31	7.82	1.67	14.73
31	55	22.96	4.9	31.4	2.6	21.9	5.0	312.3	38.8	177.8	95.8	4.29	7.79	1.66	14.67
32	54.99	23.27	4.9	31.3	2.6	22.3	5.0	316.4	38.7	181.5	96.3	4.30	7.81	1.66	14.71
33	54.99	23.15	4.9	31.3	2.6	22.1	5.0	316.9	38.7	180.0	96.1	4.30	7.81	1.66	14.69
34	55	23.08	4.9	31.4	2.6	22.0	5.0	313.9	38.7	179.2	96.0	4.30	7.80	1.66	14.68
35	54.99	23.27	4.9	31.3	2.6	22.2	5.0	316.5	38.7	181.5	96.3	4.30	7.81	1.66	14.71
36	55	22.89	4.9	31.4	2.6	21.8	5.0	311.3	38.8	176.8	95.7	4.29	7.79	1.66	14.66
38	55.01	22.72	4.9	31.4	2.6	21.5	5.0	309.0	38.8	174.8	95.5	4.28	7.77	1.65	14.62
39	54.99	23.21	4.9	31.3	2.6	22.1	5.0	315.7	38.7	180.7	96.2	4.30	7.81	1.66	14.70
40	55	22.88	4.9	31.4	2.6	21.7	5.0	311.1	38.8	176.7	95.7	4.29	7.78	1.66	14.65
41	55.01	22.7	4.9	31.4	2.6	21.5	5.0	308.7	38.8	174.5	95.4	4.28	7.77	1.65	14.62
42	55.02	22.47	4.9	31.5	2.6	21.2	5.1	305.6	38.8	171.7	95.1	4.28	7.75	1.65	14.58
43	54.99	23.13	4.9	31.4	2.6	22.0	5.0	314.5	38.7	179.7	96.1	4.30	7.80	1.66	14.69
44	54.97	23.43	4.8	31.2	2.6	22.8	4.9	318.7	38.8	183.6	96.4	4.32	7.85	1.67	14.77
45	54.99	23.13	4.9	31.6	2.6	22.0	5.0	314.5	38.7	179.7	96.1	4.30	7.80	1.66	14.69
46	55.01	22.47	4.9	31.5	2.6	21.2	5.1	305.7	38.8	171.8	95.1	4.28	7.75	1.65	14.58
47	55.01	22.66	4.9	31.4	2.6	21.4	5.1	308.2	38.8	174.1	95.4	4.28	7.76	1.65	14.61
48	55.02	22.41	4.9	31.5	2.6	21.1	5.1	304.8	38.8	171.0	95.0	4.27	7.74	1.65	14.57
49	54.99	23.12	4.9	31.4	2.6	22.0	5.0	314.5	38.7	179.7	96.1	4.30	7.80	1.66	14.69
50	55	22.85	4.9	31.4	2.6	21.7	5.0	310.8	38.8	176.4	95.6	4.29	7.78	1.66	16.65

Note: DM, dry matter; CP, crude protein; EE, ether extract; NDF, neutral detergent fiber
